# The Bacterial Cytoskeleton Modulates Motility, Type 3 Secretion, and Colonization in *Salmonella*

**DOI:** 10.1371/journal.ppat.1002500

**Published:** 2012-01-26

**Authors:** David M. Bulmer, Lubna Kharraz, Andrew J. Grant, Paul Dean, Fiona J. E. Morgan, Michail H. Karavolos, Anne C. Doble, Emma J. McGhie, Vassilis Koronakis, Richard A. Daniel, Pietro Mastroeni, C. M. Anjam Khan

**Affiliations:** 1 Centre for Bacterial Cell Biology, Institute for Cell and Molecular Biosciences, The Medical School, University of Newcastle, Newcastle, United Kingdom; 2 Department of Veterinary Medicine, University of Cambridge, Cambridge, United Kingdom; 3 Department of Pathology, University of Cambridge, Cambridge, United Kingdom; Roslin Institute, United Kingdom

## Abstract

Although there have been great advances in our understanding of the bacterial cytoskeleton, major gaps remain in our knowledge of its importance to virulence. In this study we have explored the contribution of the bacterial cytoskeleton to the ability of *Salmonella* to express and assemble virulence factors and cause disease. The bacterial actin-like protein MreB polymerises into helical filaments and interacts with other cytoskeletal elements including MreC to control cell-shape. As *mreB* appears to be an essential gene, we have constructed a viable Δ*mreC* depletion mutant in *Salmonella*. Using a broad range of independent biochemical, fluorescence and phenotypic screens we provide evidence that the *Salmonella* pathogenicity island-1 type three secretion system (SPI1-T3SS) and flagella systems are down-regulated in the absence of MreC. In contrast the SPI-2 T3SS appears to remain functional. The phenotypes have been further validated using a chemical genetic approach to disrupt the functionality of MreB. Although the fitness of Δ*mreC* is reduced *in vivo*, we observed that this defect does not completely abrogate the ability of *Salmonella* to cause disease systemically. By forcing on expression of flagella and SPI-1 T3SS *in trans* with the master regulators FlhDC and HilA, it is clear that the cytoskeleton is dispensable for the assembly of these structures but essential for their expression. As two-component systems are involved in sensing and adapting to environmental and cell surface signals, we have constructed and screened a panel of such mutants and identified the sensor kinase RcsC as a key phenotypic regulator in Δ*mreC*. Further genetic analysis revealed the importance of the Rcs two-component system in modulating the expression of these virulence factors. Collectively, these results suggest that expression of virulence genes might be directly coordinated with cytoskeletal integrity, and this regulation is mediated by the two-component system sensor kinase RcsC.

## Author Summary

*Salmonella* are major global pathogens responsible for causing food-borne disease. In recent years the existence of a cytoskeleton in prokaryotes has received much attention. In this study the *Salmonella* cytoskeleton has been genetically disrupted, causing changes in morphology, motility and expression of key virulence factors. We provide evidence that the sensory protein RcsC detects changes at the cell surface caused by the disintegration of the bacterial cytoskeleton and modulates expression of key virulence factors. This study provides insights into the importance of the integrity of the bacterial cytoskeleton in the ability of *Salmonella* to cause disease, and thus may provide a novel target for antimicrobial drugs or vaccines.

## Introduction

Salmonellae remain major global pathogens causing a broad spectrum of disease ranging from gastroenteritis to typhoid fever [1], [2]. The emergence of multidrug resistant salmonellae is complicating the management of disease [3], [4]. Hence, there is an urgent need to identify novel bacterial targets for the development of new antimicrobial agents or vaccines to combat infection.

The view that bacteria do not possess a cytoskeleton has radically changed in recent years with the discovery of intracellular filamentous protein assemblies with cell-shape defining function [5]. Although there is little primary sequence identity between eukaryotic cytoskeletal proteins and those in prokaryotes, proteins with actin- and tubulin-like structural motifs have been identified in bacteria. Bacterial cytokinesis is dependent on FtsZ which contains a structural fold mirroring tubulin. FtsZ displays similar dynamic properties to tubulin and is able to polymerise unidirectionally in a GTP-dependent manner to produce polymeric filaments [6], [7]. Polymers of FtsZ are able to assemble into a transient helical structure and subsequently form a ring-like structure around the circumference of the mid-cell [8]. This Z-ring is required for recruiting proteins for the assembly of the cell division complex [8]. The intermediate filament-like protein crescentin determines the vibroid shape of *Caulobacter crescentus* cells [9].

The bacterial proteins MreB, Mbl, and ParM display the structural and dynamic properties of eukaryotic actin [10]. Amongst these proteins, MreB is the most homologous to actin in terms of primary sequence, structure, and size [11], [12]. The most conserved region of this actin-superfamily is the ATPase domain. MreB can polymerise into helical filamentous structures important for cell morphology. Live cell microscopy in *Bacillus subtilis* revealed that MreB forms large cables which follow a helical path close to the cytoplasmic membrane [5]. An equivalent MreB protein has been found in *Escherichia coli*. When MreB is depleted, rod-shaped *B. subtilis* and *E. coli* cells become spherical [5], [13]–[15]. In *C. crescentus* MreB has been implicated to play a role in the control of cell polarity [16]. In rod-shaped bacteria the MreB polymeric structures control the localisation of cell wall growth by providing a scaffold for enzymes involved in cell wall assembly [17].

The MreB operon in *E. coli* and *B. subtilis* encodes for a number of additional genes, which do not possess any similarity to actin [18]. These include the cellular membrane proteins MreC and MreD, which also have a helical disposition. MreC forms a dimer and interestingly in *C. crescentus* MreC is localised in spirals in the periplasm [19]. Recent studies by Rothfield and colleagues provide convincing evidence to suggest that in *E. coli* MreB, MreC and MreD form helical structures independently of each other [20]. Using affinity purification and bacterial two hybrid assays, MreC and MreD appear to interact together [13]. In *E. coli* there is evidence to suggest that MreB interacts with MreC, but this may not be the case in *Rhodobacter sphaeroides* or *C. crescentus* [21]. As well as playing a key role in cell morphogenesis, MreB also has a pivotal function in chromosome segregation [22]–[24]. Adding the MreB inhibitor A22 [*S*-(3,4-Dichlorobenzyl) isothiourea] to synchronised cultures of *C. crescentus* inhibited segregation of GFP-tagged chromosomal origins [22]. However MreB may not function in chromosome segregation in *Bacillus* [15]. Recently another helically distributed cytoplasmic membrane protein which interacts with MreB named RodZ has been identified [25]–[27]. Cellular components including the RNA degradosome and lipopolysaccharide have also been identified to be localised in helical structures within the cell [28], [29].

In spite of these major advances in our understanding of the structure and organization of the bacterial cytoskeleton, there are major gaps in our knowledge of its role in bacterial pathogenicity. In this study we wished to gain insights into understanding the function of the bacterial cytoskeleton in the pathogenicity of *Salmonella*.

## Materials and Methods

### Ethics Statement

The *in vivo* experiments were covered by a Project License granted by the Home Office under the Animal (Scientific Procedures) Act 1986. This license was approved locally by the University of Cambridge Ethical Review Committee.

### Culture Conditions

*S*. Typhimurium SL1344 and mutant derivatives used in this study are described in Table 1. Strains were routinely grown in Luria-Bertani (LB) broth with appropriate antibiotics at the following concentrations: (kanamycin 50 µg ml^−1^), ampicillin (100 µg ml^−1^ or 30 µg ml^−1^ for pNDM220). A22 (Calbiochem) was added at 10 µg ml^−1^. Bacteria were grown overnight in 5 ml LB, before 25 µl of culture was used to inoculate 25 ml of fresh LB in a 250 ml flask and grown at 37°C shaking (200 rpm) unless otherwise stated. Δ*mreC* was maintained in media containing 100 µM IPTG, however for phenotypic testing this was removed unless otherwise mentioned.

**Table 1:** Strains and plasmids.

Strain	Genotype	Reference
SL1344	Parent Strain	[69]
Δ*mreC1*	SL1344 *mreC::kan*	This work
Δ*mreD1*	SL1344 *mreD::kan*	This work
Δ*mreC*	SL1344 *mreC::kan pTK521*	This work
Δ*mreD*	SL1344 *mreD::kan pTK521*	This work
ΔSPI-1	RM69 SPI-1::kan	[70]
ΔSPI-2	12023 *ssaV::kan*	[71]
ΔflhDC	LT2 *flhDC::kan*	[72]
Δ*rcsA*	SL1344 *rcsA::kan*	This work
Δ*rcsB*	SL1344 *rcsB::kan*	This work
Δ*rcsC*	SL1344 rcsC::cat	This work
Δ*rcsD*	SL1344 *rcsD::kan*	This work
Δ*rcsF*	SL1344 *rcsF::kan*	This work
Δ*rcsDB*	SL1344 *rcsDB::kan*	This work
Δ*rcsCBD*	SL1344 *rcsCBD::kan*	This work
Δ*mreC* Δ*rcsA*	SL1344 *mreC::cat rcsA::kan*	This work
Δ*mreC* Δ*rcsB*	SL1344 *mreC::cat rcsB::kan*	This work
Δ*mreC* ΔrcsC	SL1344 *mreC::kan rcsC::cat*	This work
Δ*mreC* Δ*rcsD*	SL1344 *mreC::cat rcsD::kan*	This work
Δ*mreC* Δ*rcsDB*	SL1344 *mreC::kan rcsDB::cat*	This work
Δ*mreC* Δ*rcsCBD*	SL1344 *mreC::cat rcsCBD::kan*	This work
Δ*mreC* Δ*rcsF*	SL1344 *mreC::cat rcsF::kan*	This work
Δ*mreC* Δ*qseF*	SL1344 *mreC::kan qseF::cat*	This work
Δ*mreC* Δ*phoBR*	SL1344 *mreC::kan phoBR::cat*	This work
Δ*mreC* Δ*yjiGH*	SL1344 *mreC::kan yjiGH::cat*	This work
Δ*mreC* Δ*baeSR*	SL1344 *mreC::kan baeSR::cat*	This work
Δ*mreC* Δ*basSR*	SL1344 *mreC::kan basSR::cat*	This work
Δ*mreC* Δ*hydH*	SL1344 *mreC::kan hydH::cat*	This work
Δ*mreC* Δ*qseBC*	SL1344 *mreC::kan qseBC::cat*	This work
Δ*mreC* Δ*tctDE*	SL1344 *mreC::kan tctDE::cat*	This work
Δ*mreC* Δ*cpxAR*	SL1344 *mreC::*kan cpxAR::cat	This work
YVM004	SJW1103 *gfp-fliG*	[32]
YVM004 *mreC*	SJW1103 *gfp-fliG mreC::kan*	[32]
TH3724	P*flhDC*::T-POP (DEL-25) *flhC*5213::MudJ	[33]

Plasmid	Description	Reference
pBR322	Cloning vector	[73], [74]
pBAD24	Cloning vector	[75]
pZEP08	GFP+ transcriptional fusion vector	[76]
pBR322-*gfp*	pBR322 with *gfp*	This work
pBR322-*mreBgfp*	pBR322*gfp* with *mreB*	This work
pNDM220	Low copy cloning vector	[77]
pNDM220-*mreBgfp*	*mreBgfp* subcloned from pBR322-*mreBgfp*	This work
pKD13	Lambda Red template	[31]
pKD46	Lambda Red recombinase	[31]
pLE7	*gfpmreB*	[36]
pTK521	pNDM220 pA1/O4/O3::*mreBCD*	[14]
pCS26	*luxCDABE* promoter reporter vector	[49]
pCS26-*hilA*	*hilA*	[49]
pCS26-*hilC*	*hilC*	[49]
pCS26-*hilD*	*hilD*	[49]
pSB401	*luxCDABE* promoter reporter vector	[41]
pBA409	*sopB* promoter reporter	[41]
pRG34	pSB401-*fliA*	[41]
pRG38	pSB401-*flhD*	[41]
pRG46	pSB401-*fliC*	[41]
pRG51	pSB401-*flgA*	[41]
pMK1-*lux*	*luxCDABE* promoter reporter vector	[52]
pMK1-*lux*-*ssaG*	pMK1-*lux*-*ssaG*	This work
pBAD24-*hilA*	*hilA* inducible expression plasmid	This work
pBAD24-*rcsC*	*rcsC* complementation plasmid	This work

For the SPI-1 T3S studies cells were grown overnight in LB before subculturing 1/100 into 25 ml fresh LB and growing at 37°C for approximately 5 hrs with good aeration until OD600_nm_∼1.2 in 250 ml flasks [30]. For the SPI-2 T3S studies cells were grown in SPI-2 induction media (100 mM Tris-base, 0.1% w/v casamino acids, 0.1% w/v glycerol, 10 µM MgSO_4_, 40 µg ml^−1^ histidine, pH 5.8). Cells were grown overnight in LB before subculturing 1/100 in 25 ml SPI-2 inducing media before growing for 16 h at 37°C in 250 ml flasks before sampling.

### Motility Assays

Cells were inoculated from a fresh LB plate onto the semi-solid motility agar (10 g l^−1^ Bacto-tryptone, 5 g l^−1^ NaCl, 3 g l^−1^ agar) and incubated upright for a minimum of 5 h. Distinct zones of cell motility were measured and compared to WT SL1344 and non-motile SL1344 strains.

### Chromosomal Gene Disruptions and Depletion Mutants

Chromosomal gene deletions were constructed using the lambda Red method as described previously [31], before transducing the mutation into a genetically clean parent strain using bacteriophage P22*int*. In the case of Δ*mreC* and Δ*mreD* the mutations were transduced into a parent strain containing pTK521 (*lac-mreBCD E. coli*) to complement the mutation in the presence of 100 µM isopropyl beta-D-1-thiogalactopyranoside (IPTG). Gene deletion primers typically encompassed the first and final 20 bases of the coding sequence of the respective gene were synthesised. However, as the *mreC* and *mreD* gene coding sequences overlap by a single base, to ensure only a single coding sequence was disrupted the respective *mreC* 3′ primer and *mreD* 5′ primer were moved internally into their coding sequence such as to produce no overlapping mutations. Gene deletions for the two-component systems (Δ*qseF,* Δ*phoBR*, Δ*yjiGH*, Δ*baeSR*, Δ*basSR*, Δ*hydH*, Δ*qseBC* Δ*tctDE,* Δ*cpxAR,* Δ*rcaA,* Δ*rcsB,* Δ*rcsC,* Δ*rcsD,* Δ*rcsDB, and* Δ*rcsCBD*), were constructed in SL1344 WT using classical lambda Red methods before transducing into the Δ*mreC* strain using bacteriophage P22*int*. Primers are listed in Table 2.

**Table 2:** PCR primers.

Primer	Sequence (5′-3′)
*mreC-*P1	GGATTGTCCTGCCTCTCCGACGCGAGAATACGCATAGCCTGTGTAGGCTGGAGCTGCTTC
*mreC-*P4	CATCAGGCGCTCATTGGCGACGCGGTGAACCTCTTCCGGATTCCGGGGATCCGTCGACC
*mreC*5	ATACGGGCAGGATTATCCCT
*mreC*3	GCGCAATAAGAAACGAGAGC
*mreD-*P1	GGCGCGACCACGCCGCCTGCGCGTGCGCCGGGAGGGTAGTGTAGGCTGGAGCTGCTTC
*mreD-*P4	GGGGAACCGGAAGCAAGATACAGAGTTGTCATATCGACCTATTCCGGGGATCCGTCGACC
*mreD*5	ATCAACGCAACCATCGCCTT
*mreD*3	TCAATAATTCCTGGCGACGC
*qseBC* P1	GTTAACTGACGGCAACGCGAGTTACCGCAAGGAAGAACAGGTGTAGGCTGGAGCTGCTTC
*qseBC* P4	AAATGTGCAAAGTCTTTTGCGTTTTTGGCAAAAGTCTCTGATTCCGGGGATCCGTCGACC
*qseBC* 5′	ACATCGCCTGCGGCGACAAG
*qseBC* 3′	GCGGTGCGGTGAAATTAGCA
*rcsA* P1	GTAAGGGGAATTATCGTTACGCATTGAGTGAGGGTATGCCGTGTAGGCTGGAGCTGCTTC
*rcsA* P4	AATTGAGCCGGACTGGAGGTACATTGCCAGTCCGGATGTCATTCCGGGGATCCGTCGACC
*rcsA* 5′	GATTATGGTGAGTTATTCAG
*rcsA* 3′	CGAGAAGGCGGAGCAGGACT
*rcsB* P1	GCCTACGTCAAAAGCTTGCTGTAGCAAGGTAGCCCAATACGTGTAGGCTGGAGCTGCTTC
*rcsB* P4	ATAAGCGTAGCGCCATCAGGCTGGGTAACATAAAAGCGATATTCCGGGGATCCGTCGACC
*rcsB* 5′	CGTGAGAAAGATGCTCCAGG
*rcsB* 3′	TGAGTCGACTGGTAGGCCTG
*rcsC* P1	GTCACACTCTATTTACATCCTGAGGCGGAGCTTCGCCCCTGTGTAGGCTGGAGCTGCTTC
*rcsC* P4	TTTTACAGGCCGGACAGGCGACGCCGCCATCCGGCATTTTATTCCGGGGATCCGTCGACC
*rcsC* 5′	CGTCATTTACCGCTACCTTA
*rcsC* 3′	GGCCTACCAGTCGACTCATC
*rcsD* P1	CCTTCACCTTCAGCGTTGCTTTTACAGGTCGTAAACATAAGTGTAGGCTGGAGCTGCTTC
*rcsD* P4	ACCTTGCTACAGCAAGCTTTTGACGTAGGCGTCAATGTCGATTCCGGGGATCCGTCGACC
*rcsD* 5′	TTCATTACCCTTTATACTGC
*rcsD* 3′	CATATTGTTCATGTATTGGG
*rcsF* P1	TTCAATATCTGGCAATTAGAACATTCATTGAGGAAATATTGTGTAGGCTGGAGCTGCTTC
*rcsF* P4	GGGGAGCGAATAACGCCGATTTGATCAAACTGAAAGCTGCATTCCGGGGATCCGTCGACC
*rcsF* 5′	TCATTTATGCAAGCTCCTGA
*rcsF* 3′	CGGCGAATTTTTCTTTATAG
*rcsCBD* P1	GTCACACTCTATTTACATCCTGAGGCGGAGCTTCGCCCCTGTGTAGGCTGGAGCTGCTTC
*rcsCBD* P4	CCTTCACCTTCAGCGTTGCTTTACAGGTCGTAAACATAAATTCCGGGGATCCGTCGACC
*rcsCBD* 5′	CGTCATTTACCGCTACCTTA
*rcsCBD* 3′	TTCATTACCCTTTATACTGA
*rcsDB* P1	CCTTCACCTTCAGCGTTGCTTTTACAGGTCGTAAACATAAGTGTAGGCTGGAGCTGCTTC
*rcsDB* P4	ATAAGCGTAGCGCCATCAGGCTGGGTAACATAAAAGCGATATTCCGGGGATCCGTCGACC
*rcsDB* 5′	TTCATTACCCTTTATACTGC
*rcsDB* 3′	TGAGTCGACTGGTAGGCCTG
*phoBR* P1	ATGGCGCGGCATTGATAACTAACGACTAACAGGGCAAATTGTGTAGGCTGGAGCTGCTTC
*phoBR* P4	CATCCGCTGGCTTATGGAAAGTTATACTTACGAAAGGCAAATTCCGGGGATCCGTCGACC
*phoBR* 5′	TGTCATAAATCTGACGCATA
*phoBR* 3′	CTGCAAAGAAAATAAGCCAG
*qseF* P1	GGCGCCGTCGCCGTCACAAGATGAGGTAACGCCATGATAAGTGTAGGCTGGAGCTGCTTC
*qseF* P4	TTAAACGTAACATATTTCGCGCTACTTTACGGCATGAAAAATTCCGGGGATCCGTCGACC
*qseF* 5′	CAAACCCGCGACGTCTGAAG
*qseF* 3′	GTCGCCTGTGTTTTGATCGG
*cpxAR* P1	CGCCTGATGACGTAATTTCTGCCTCGGAGGTACGTAAACAGTGTAGGCTGGAGCTGCTTC
*cpxAR* P4	CGAGATAAAAAATCGGCCTGCATTCGCAGGCCGATGGTTTATTCCGGGGATCCGTCGACC
*cpxAR* 5′	GTAAAGTCATGGATTAGCGA
*cpxAR* 3′	CTCCCGGTAAATCTCGACGG
*tctDE* P1	AATTCCCTTTCAATGCGGCAGAAACTTTACAGGATGTGATGTGTAGGCTGGAGCTGCTTC
*tctDE* P4	TTTTTGTAAACGTGCTTTACCGCTGACACATTTGTCCGCAATTCCGGGGATCCGTCGACC
*tctDE* 5′	TGTTAAAACAATAACCTTTC
*tctDE* 3′	GTCACACCTCAAGATGCGAC
*yjiGH* P1	TTCCTGCTCCCAGCTCCGGCCTGCGTCAACACCTGTTTCTGTGTAGGCTGGAGCTGCTTC
*yjiGH* P4	TAAACTCCGCGGCGGATAAATCAGGCATGATAACTCCTTAATTCCGGGGATCCGTCGACC
*yjiGH* 5′	TCAAATTTATTTCTCCTTTT
*yjiGH* 3′	GTGCGCACCCTGTAATAAGG
*HydH* P1	TCTGGTTGCCAGTGATAGCGAGACAACAGGATTAACAAGGGTGTAGGCTGGAGCTGCTTC
*HydH* P4	GTAACGACATTGGCTGGCGCGCCATTGAGCGTGAGCAAAAATTCCGGGGATCCGTCGACC
*HydH* 5′	TAAAGGCGCGGTCTTTACTA
*HydH* 3′	CTGGGACGGCAGCTTCAGCC
*BasS* P1	CTACATGCTGGTTGCCACTGAGGAAAGCTAAGTGAGCCTGGTGTAGGCTGGAGCTGCTTC
*BasS* P4	AGTTTTATCTATGTGTGGGTCACGACGTATTAAACGCCTGATTCCGGGGATCCGTCGACC
*BasS* 5′	CGCACGGTTCGCGGGTTTGG
*BasS* 3′	GTAGTGTGCTGATTGTCAGC
*BaeSR* P1	TGGTCATTTCACGGCGTAAAAGGAGCCTGTAATGAAAGTCGTGTAGGCTGGAGCTGCTTC
*BaeSR* P4	ATATCGTCTTACGACCTTGTTATTGTTATGCCAATAATCAATTCCGGGGATCCGTCGACC
*BaeSR* 5′	CCGCGTGCCGAACGATACAC
*BaeSR* 3′	CAGAATAGCGTTGGCGGAAA
pEGFP5	GCG**GAATTC**AGGTACCCCCGGGCCATGGTCTAGAATGGTGAGCAAGGGCGAGG
pEGFP3	GCG**AAGCTT**TTACTTGTACAGCTCGTCC
*mreB*5 Eco	GCG**GAATTC**GCAGATGTTTGTCAACACATC
*mreB*3 Xba	GCG**TCTAGA**CTCTTCGCTGAACAGGTCGCC
ssaG5 Eco	GCG**GAATTC**CGACAGTATAGGCAATGCCG
ssaG3 Bam	GCG**GGATCC**CCACTAATTGTGCAATATCC
BAD*hilA*5	GCG**GAATTC**ATGCCACATTTTAATC
BAD*hilA*3	GCG**TCTAGA**TTACCGTAATTTAATC
BAD*rcsC*5	GCG**GAATTC**TTGAAATACCTTGCTTC
BAD*rcsC*3	GCG**AAGCTT**TTATGCCCGCGTTTTACGTACCC

Bold indicates restriction enzyme recognition sites.

### Construction of the MreB-GFP Fusion Vector

GFP was amplified from pZEP08 and cloned along with a new multiple cloning site into the *Eco*RI and *Hin*dIII sites of pBR322 to create pBR322GFP. *mreB* along with its natural promoter was amplified from genomic DNA and cloned into the *Eco*RI and *Xba*I sites of pBR322GFP, before the *mreB-gfp* fusion was subcloned from the pBR322*mreB-gfp* into pNDM220 using the *Eco*RI and *Bam*HI sites.

### Transcriptional Reporter Fusions

Flagella and SPI1 transcriptional reporter plasmids were transformed into SL1344 and Δ*mreC* mutant cells. Expression from the *lux* transcriptional reporters was measured during the growth cycle of 10^−3^ diluted overnight cultures cells grown in microtitre plates (200 µl total volume) for a minimum of 15 h at 37°C with periodic shaking. Optical density (600_nm_) and relative luminescence was measured at 15 minute intervals using a Tecan Infinity200 luminometer. Samples were tested in triplicate, and repeated at least 3 times.

### Construction of Complementation Plasmids

The *hilA* and *rcsC* open reading frames were amplified from SL1344 genomic DNA and cloned into the *Eco*RI and *Xba*I or the *Eco*RI and *Hind*III sites of pBAD24 to create pBAD*hilA* and pBAD*rcsC* respectively.

### Protein Manipulation

Whole cell total protein samples were obtained by pelleting an appropriate volume of bacterial culture, followed by resuspension in SDS-loading buffer and boiling for 10 mins. Culture supernatants were filter sterilized (0.22 µm) and proteins were ammonium sulphate precipitated (4 g 10 ml^−1^ supernatant) overnight at 4°C. Precipitated secreted proteins were resuspended in H2O and then combined with an equal volume of sample buffer (Biorad). Whole cell and culture supernatant samples were run on 12% SDS/PAGE and transferred on Protran nitrocellulose transfer membranes (Schleicher & Schuell) using a wet transfer apparatus (Biorad). Western blot analysis was performed using polyclonal SipA, SipB, SipC or PrgH for testing SPI-1 T3S functionality, coupled with a goat anti-mouse horseradish peroxidase-labelled secondary antibody (Dako Cytomation). Detection was carried out using 4-chloro-1-naphthol (Sigma) according to the manufacturer's instructions.

### *In vivo* Inoculation and Growth Curves

Female C57BL/6 mice were purchased from Harlan Olac Ltd., (Blackthorn, Bicester, UK). Mice were used when over eight weeks of age. Bacterial suspensions for injection were grown for 16 h as a stationary culture at 37oC in LB broth. Bacteria were diluted in PBS prior to injection into a lateral tail vein. Mice were killed by cervical dislocation and the livers and spleens aseptically removed. Each organ was homogenised (separately) in a Seward Stomacher 80 Biomaster (Seward) in 10 ml of distilled water and viable bacterial counts in the homogenate were assayed on pour plates of LB agar. Representative bacterial colonies were kept and re-tested for phenotypic changes.

### Construction of Flagella Live Cell Imaging Strains

Wild type *Salmonella* SJW1103 cells with chromosomal N-terminal GFP fusion to *fliG* (YVM004) [32] were P22 transduced with the *mreC::kan* mutation to create YVM004 Δ*mreC*. This strain, along with the WT control, was subsequently transduced with a chromosomally-based inducible *flhDC* locus derived from TH2919 [33].

### Visualisation of Type 3 Secretion Systems and Flagella

Cells were grown to the appropriate growth phase (mid-log for SPI-1 and flagella, or stationary phase for SPI-2) in relevant media (LB or SPI-2 inducing media). Flagella visualisation strains (*fliG-gfp*), were mounted on 1% agarose beds for imaging. Samples for visualising the type 3 secretion apparatus were fixed in 4% paraformaldehyde diluted in PBS for 1 h before washing for 15 minutes in three changes of PBS. Samples were incubated with either αSipA, αSipB, αSipC, αSipD (SPI-1) or αSseB (SPI-2) antibodies diluted 1∶1000 in PBS for 3 h with gentle agitation. Samples were subsequently washed in PBS before incubating in 1∶1000 Alexa Fluor 488 conjugated goat anti-rabbit antibody (Invitrogen-Molecular Probes, Paisley, U.K.), washed for 30 mins in fresh PBS before mounting onto agarose beds.

### Tissue Immunostaining for Fluorescence Microscopy

Half of each organ was fixed overnight in 4% paraformaldehyde diluted in PBS, washed for 90 min in three changes of PBS and then immersed in 20% sucrose (in PBS) for 16 h at 4oC before being embedded in Optimal Cutting Temperature (OCT) (Raymond A Lamb Ltd, Eastbourne, U.K.) in cryomoulds (Park Scientific, Northampton, U.K.). Samples were frozen and stored at -80oC. 30 µm sections were cut, blocked and permeabilised for 10 min in a permeabilising solution containing 10% normal goat serum and 0.02% Saponin in PBS (Sigma, Poole, UK). Sections were stained with 1∶1000 dilution of rat anti-mouse CD18^+^ monoclonal antibody (clone M18/2, BD Pharmingen), together with a 1∶500 dilution of rabbit anti-LPS O4 agglutinating serum (Remel Europe Ltd), for 16 h at 4oC. Subsequently, sections were washed in PBS then incubated with 1∶200 Alexa Fluor 568-conjugated goat anti-rat antibody (Invitrogen-Molecular Probes, Paisley, U.K.) and a 1∶1000 dilution of Alexa Fluor 488-conjugated goat anti-rabbit antibody (Invitrogen-Molecular Probes, Paisley, U.K.). All sections were mounted onto Vectabond-treated glass slides (Vector Laboratories Ltd.) using Vectashield containing DAPI (Vector Laboratories Ltd.).

### Microscopy

All phase contrast and fluorescence images were captured using an Andor iXon^EM^+ 885 EMCCD camera coupled to a Nikon Ti-E microscope using a 100x/NA 1.4 oil immersion objective. Images were acquired with NIS-ELEMENTS software (Nikon) and processed using ImageJ. Fluorescence images were deconvolved using Huygens Deconvolution software (Scientific Volume Imaging). Cell measurements were taken on a Nikon Ti-E microscope with NIS-ELEMENTS software. Immunofluorescence images from tissue sections were analysed multi-colour fluorescence microscopy (MCFM) using a Leica DM6000B Fluorescence microscope running FW4000 acquisition software.

### Transepithelial Resistance and Bacterial Effector Translocation Assays

The effect of *Salmonella* infection on transepithelial resistance (TER) was determined for differentiated Caco-2 cells as previously described [34]. Briefly, the Caco-2 cells were grown on transwell inserts (Corning, UK) until differentiated (12–14 days), before the transepithelial resistance was measured for each well. *Salmonella* strains were then added to the cells at a multiplicity of infection (MOI) of 20, and the cells incubated for 4 h. TER measurements were taken every hour and the results given as a ratio of TER (t)/(t^0^) to show the percentage change in TER over the course of the experiment. Data were collated and analysed for statistical differences (Student's t-test) in Minitab.

Samples for the assay of translocated effector proteins were isolated from differentiated Caco-2 cells grown in 6 well plates after infection with an MOI of 20 for 4 h. Excess bacteria were washed off before the cells were solubilised in 0.01% Triton X-100 and centrifuged to remove bacteria and host cell membranes. The host cell cytoplasmic fractions were analysed by western blotting with αSipB antibody.

## Results

### *In silico* Identification of the *Salmonella* Actin Homologue *mreB*

We wished to identify and characterise putative *Salmonella* cytoskeletal gene homologues. A BLAST search of the *S.* Typhimurium genome sequence database (www.ncbi.nlm.nih.gov) [35] for the known *E. coli* actin-homologue MreB identified a putative *mre* operon of high sequence identity. Comparison of the *Salmonella* genes to those of *E. coli* showed 100% (*mreB*), 88% (*mreC*) and 94% (*mreD*) homology at amino acid level, comparisons of these same genes to those in *B. subtilis* revealed sequence homologies of 57%, 24% and 27% respectively.

### MreB Proteins Are Helically Localised

**Figure 1:**
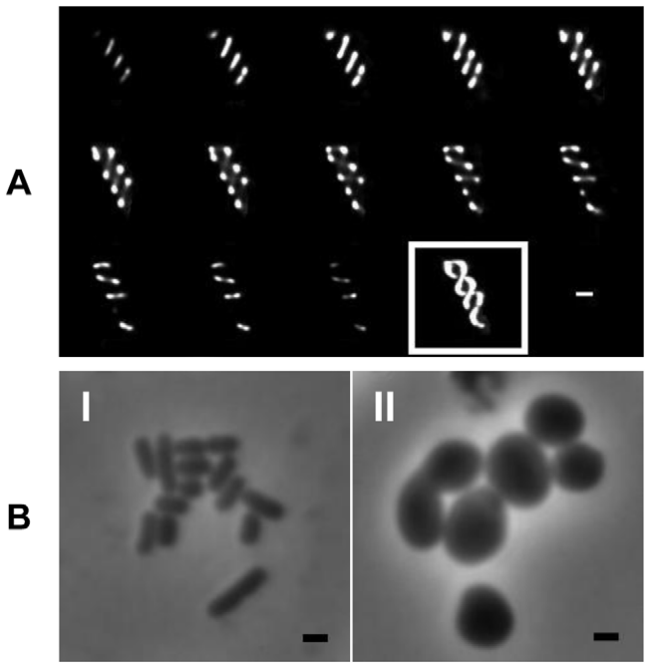
Localization and morphological role of the *S.* Typhimurium Mre proteins. (A) Fluorescence microscopy montage showing z-sections taken of MreB-GFP fusions in WT SL1344 revealing a helical distribution. Slices taken at 0.1 µm intervals on live cells in mid log phase going from left to right followed by maximum intensity projection (boxed). (B) Morphology of WT *S.* Typhimurium (I) and Δ*mreC* (II) reveal the mutant has changed from rod to round-shaped, with some heterogeneity in size noted. In all images the bar represents 1 µm.

In order to determine the localisation of MreB in *Salmonella*, vectors expressing N and C terminal fusions of MreB to GFP were used. The N-terminal fusion plasmid has already been described [36], and we constructed a C-terminal fusion vector. Both constructs revealed a helical distribution of MreB along the long axis of the cell. The helices were discerned by assembling a series of z-stack images taken in successive planes by using Metamorph imaging and Huygens deconvolution software (Figure 1A).

### Construction of *mre* Mutants

The *mreB* gene appears to be essential in bacteria including *Salmonella* (data not shown), and Δ*mreB* viable cells often contain compensatory mutations [37]. Each of the components of the cytoskeletal complex, for example MreB, MreC, or MreD, are essential for its function. As an alternative strategy to study the function of the cytoskeleton we therefore generated a *mreC* depletion strain under conditions designed to minimise selective pressures for undefined secondary compensatory mutations [37]. Using the lambda Red one-step gene disruption method, we successfully constructed a *mreC::kan* mutant in the *S*. Typhimurium wild-type strain SL1344 [31]. This mutation leaves intact the first gene in the operon *mreB.* Using bacteriophage P22*int* the *mreC::kan* mutation was then transduced into a genetically “clean” SL1344 strain harbouring p*lac-mre* operon (pTK521) [14] and the resulting strain designated Δ*mreC*. The p*lac-mre* operon is a low copy number plasmid expressing the *mre* operon from the IPTG-inducible *lac* promoter. The identity of the mutation was confirmed by PCR and DNA sequencing. Expression of MreC was assessed by western blotting in the mutant strains, revealing no detectable levels MreC unless complementation was induced (Figure S1). In addition to the Δ*mreC* mutant, the lambda Red method was used to generate Δ*mreD.*

### Morphology and Growth Rates

When the morphology of the Δ*mreC* mutant was examined microscopically, the cells were no longer rod-shaped but spherical (Figure 1B). Upon the addition of IPTG the morphology of the Δ*mreC* strain was restored to the wild-type rod shape. Under microscopic examination the Δ*mreD* mutant displays a similar morphological phenotype to the Δ*mreC*. WT cells were measured to be on 1.61(+/−0.49) µm in length and 0.75(+/− 0.17) µm in width, whereas the Δ*mreC* cells were 2.03(+/−0.60) µm in length and 1.21(+/−0.41) µm in width. Complementation of the Δ*mreC* mutant with 100 µM IPTG resulted in wild type shaped cells 1.82(+/−0.44) µm in length and 0.78(+/−0.24) µm in width. Measurements were taken from a minimum of 350 cells per strain. Growth rates of the strains were determined in LB media at 37°C revealing a ∼50% increase in the lag phase of the Δ*mreC* mutants (Figure S2), which subsequently grow at a comparable rate to that of the wild type or complemented mutant strains during log phase.

### Motility and Expression of Flagellin Subunits

The motility phenotype of Δ*mreC* was examined on semi-solid agar plates. In contrast to the isogenic parent, the Δ*mreC* cells were no longer motile. Surprisingly, this motility defect has not been reported in either *E. coli* or *B. subtilis*. Cellular and secreted proteins of the parent SL1344 and Δ*mreC* were examined by SDS-PAGE and western blotting using antibodies directed against the phase-1 and phase-2 flagellin subunits FliC and FljB. Neither of these subunits were present in either the secreted or cellular proteins, explaining the inability of the cells to swim (data not shown). The non-motile phenotype was fully complementable *in trans* upon the addition of IPTG to the mutant strain harbouring pTK521 (Figure S3).

### Expression of Flagella Genes

We observed that the *Salmonella* Δ*mreC* depletion strain was non-motile and failed to express flagella subunits FliC or FljB. The regulation and assembly of flagella in *Salmonella* is complex. Flagella genes are arranged into 14 operons and their transcription is organised into a regulatory hierarchy of early (class I), middle (class II), and late genes (class III) [38]. The class I *flhDC* operon is the master regulator, with FlhD and FlhC forming a heterotetramer that is required for transcriptional activation of the class II genes, which encode the hook-basal body complexes and the alternative sigma factor FliA (sigma28). FliA alone or with FlhDC, activates expression of the class III operon genes, which encode the filament protein, hook-associated proteins, motor proteins, and chemotaxis proteins [39], [40]. The class III genes are further subdivided into *fliA*-independent expression class IIIa or class IIIb [41]. In order to systematically investigate the mechanistic basis for the Δ*mreC* motility phenotype we have taken selected class I, II, and III regulated flagella gene promoter fusions to a luciferase reporter gene, and monitored their expression by luminescence in wild type and Δ*mreC* strains. Constructs with *flhD* (class I*)*, *fliA, flgA*, (class II), and *fliC* (class III) promoters fused to the luciferase reporter gene were used. The reporter plasmid pSB401 has a promoterless *luxCDABE* operon and was used as a control.

**Figure 2:**
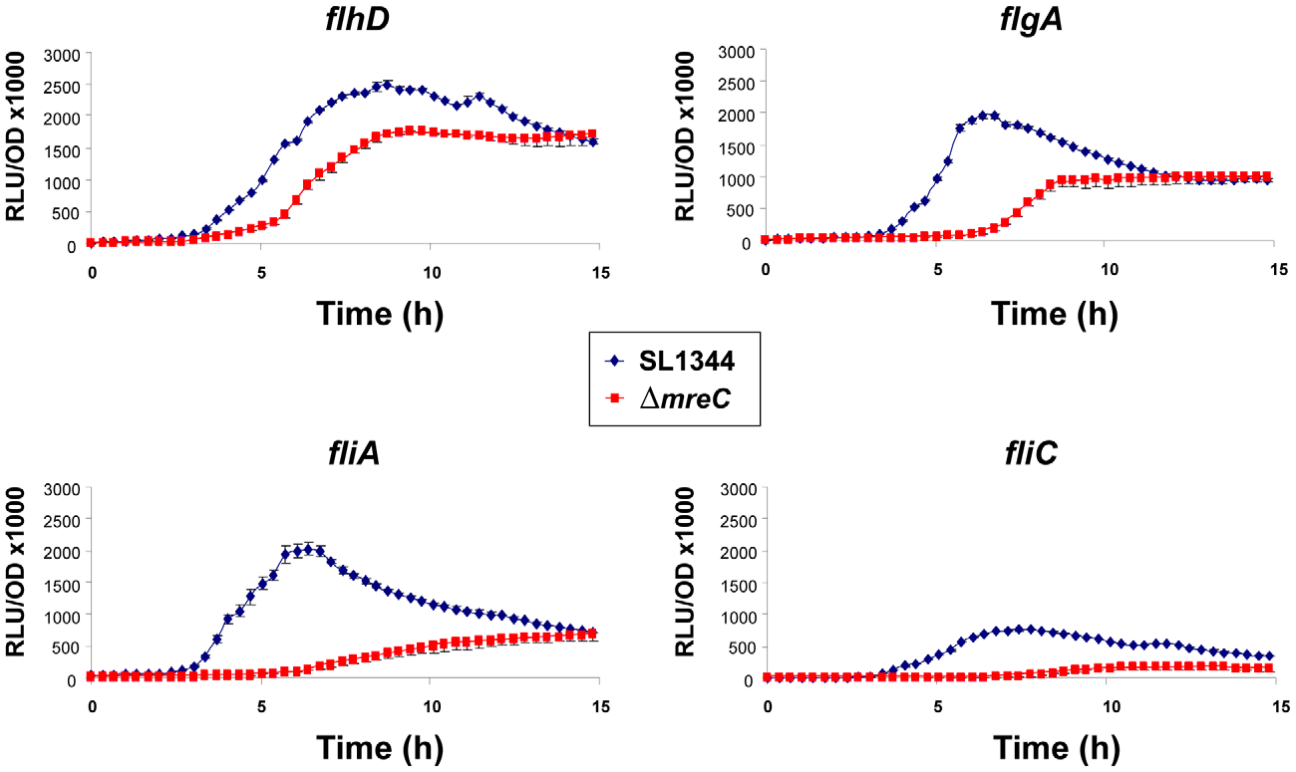
Impact of Δ*mreC* on the transcription of flagellar genes. Transcriptional expression profiles of *flhD*, *flgA*, *fliA* and *fliC* promoter reporters in WT SL1344 (blue diamonds) and Δ*mreC* (red squares) expressing the *Photorhabdus luminescens* LuxCDABE luciferase. Experiments were repeated at least three times and error bars indicate standard deviation.

The class I *flhD* promoter displayed a reduction in the level of expression in Δ*mreC* compared to the wild-type strain suggesting the class I promoter has reduced activity. Notably greater changes in the expression profiles occur in other class II and class III genes. The class II promoters for the operons encoding the transcriptional regulators *fliAZY* and *flgAM* display significant reductions in expression levels in Δ*mreC* (Figure 2). As predicted from the western blotting data expression of the *fliC* class III promoter was significantly reduced. Collectively, the promoter-reporter activity data can account for the motility defect.

### Expression of SPI-1 and SPI-2 Type 3 Secretion System Proteins

**Figure 3:**
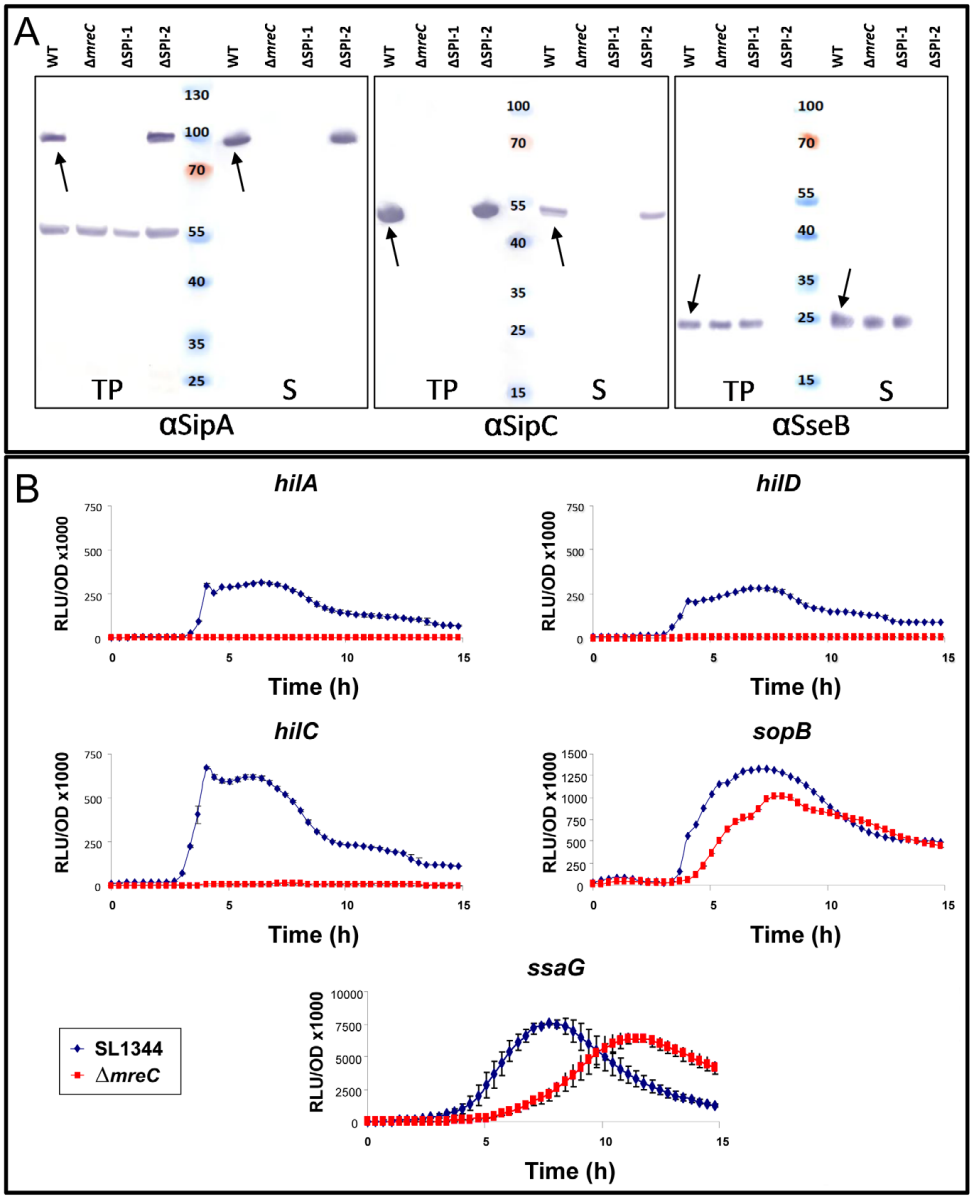
Expression of *Salmonella* SPI-1 and SPI-2 effector proteins in Δ*mre*C. (A) Expression of SPI-1 proteins in WT SL1344, Δ*mreC*, ΔSPI-1, and ΔSPI-2 mutants during SPI-1 inducing conditions as revealed by western blotting with polyclonal αSipA and αSipC antibodies. Expression of SPI-2 in WT SL1344, Δ*mreC*, ΔSPI-1, and ΔSPI-2 mutants during SPI-2 inducing conditions as revealed by western blotting of membrane fraction samples with polyclonal αSseB antibody. Samples representing total proteins and secreted proteins are shown. Arrows indicate the respective protein bands. (B)Transcriptional expression profiles of *hilA*, *hilC*, *hilD*, *sopB* (SPI-1) and *ssaG* (SPI-2) promoter reporters in WT SL1344 (blue diamonds) and Δ*mreC* (red squares). Experiments were repeated at least three times and error bars indicate standard deviation.

Type 3 secretion systems are essential for the virulence of a range of pathogens including *Salmonella* [42], [43]. The secretion apparatus assembles into a supramolecular needle-complex. Secreted effector proteins in the bacterial cytoplasm traverse through the needle-complex and the bacterial multi-membrane envelope, directly into host cells [44]–[46]. The apparatus anchors to the cell envelope via a multi-ring base. *Salmonella* possess two T3SS's encoded by pathogenicity islands (SPI's). The SPI-1 T3SS is important for invasion of intestinal epithelial cells and the SPI-2 T3SS plays a central role in survival within the hostile environment of a macrophage. The SPI-1 T3S system translocates virulence effector proteins into the cytosol of epithelial cells resulting in rearrangements of the actin cytoskeleton which enable *Salmonella* to invade [47]. To investigate whether the *mreC* mutation has an impact on SPI-1 T3S, we used western blotting to determine the presence and functionality of the system using antibodies to an apparatus protein PrgH as well as the effector proteins SipA and SipC, in both SL1344 and Δ*mreC*. In contrast to the wild-type SL1344, the T3S structural and effector proteins were not expressed in the cellular or secreted fractions from the Δ*mreC* depletion mutant (Figure 3A). This suggests that SPI-1 T3S in the Δ*mreC* mutant is not fully functional. The expression and secretion phenotypes were fully complementable *in trans* upon the addition of IPTG (data not shown).

**Figure 4:**
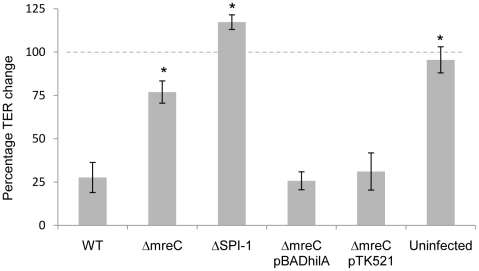
Percentage change in transepithelial resistance of differentiated Caco-2 cells after 4hr infection. TER of polarised Caco-2 monolayers exposed to *Salmonella* strains at an MOI of 20. TER change is expressed as a percentage alteration at 4hr compared to the initial value at time zero. Error bars indicate the standard deviations derived from at least three independent experiments. ^*^ Indicates statistical difference from WT (p<0.05).

The functional assembly of SPI-1 T3SS was also confirmed using transepithelial resistance (TER) assays in differentiated Caco-2 cells, showing a reduced ability to disrupt epithelial tight junctions in the Δ*mreC* mutant compared to the wild type strain (Figure 4).

To further assess the disruption of the functionality of the SPI-1 T3S, a translocation assay was performed in Caco-2 cells infected with the strains. Host cell cytoplasmic proteins were probed for the bacterial effector protein SipB using western blotting (Figure S4). This revealed the inability of the Δ*mreC* mutants to infect host epithelia and disrupt their tight junctions. In addition, Δ*mreC* was fully complementable in this assay following IPTG induction.

The SPI-2 T3SS is pivotal for the establishment of the *Salmonella* containing vacuole (SCV) inside macrophages and subsequent survival [43]. We next investigated the effect of the Δ*mreC* mutation on the functionality of the SPI-2 T3SS. The strains were grown under SPI-2 inducing conditions and the T3S of the translocon protein SseB monitored. SseB together with SseC and SseD function as a translocon for other effector proteins and SseB is normally found associated with the outer surface of *Salmonella*. Thus membrane fractions were purified to monitor expression and T3S by western blotting. This revealed that in contrast to the SPI-2 negative control (*ssaV*), SseB was secreted and associated with the bacterial membrane surface in both the wild-type and Δ*mreC* strains (Figure 3A). This provides qualitative evidence to suggest that in contrast to the SPI-1 T3SS, the SPI-2 T3SS appears to remain functional.

### Expression of SPI-1 and SPI-2 Type 3 Secretion System Regulatory Genes

Several environmental signals and transcriptional factors modulate expression of the SPI-1 T3SS. We wished to understand the mechanistic basis by which expression of the SPI1-T3SS is down-regulated. Within SPI-1 there are key transcriptional activators which regulate expression of SPI-1 genes: HilC, HilD, HilA, and InvF. Both HilC and HilD activate expression of SPI-1 genes by binding upstream of the master regulatory gene *hilA* to induce its expression[48]. HilA binds and activates promoters of SPI-1 operon genes encoding the type 3 secretory apparatus, several secreted effectors, and the transcriptional regulator InvF. InvF activates expression of effector genes inside SPI1 and also effector genes outside SPI-1 such as *sopB* and *sopE* [47].

Expression of selected SPI-1 T3SS genes was monitored using transcriptional promoter reporters in Δ*mreC*, using constructs harbouring the *hilA*, *hilC*, *hilD*, *invF* and *sopB* promoters fused to the promoterless *luxCDABE* operon that produces light in response to gene expression [49]–[51]. Each construct was introduced into both wild-type SL1344 and Δ*mreC* depletion mutant, and the level of expression of the promoters in these strains monitored by luminescence assays. WT SL1344 and Δ*mreC* cells harbouring pCS26 or pSB401 vectors alone were used as controls, and did not produce any luminescence as expected. The reporter assays revealed that the SPI-1 transcription factor gene promoters for *hilA*, *hilC*, *hilD*, and *invF* were completely inactive in Δ*mreC* in contrast to the wild-type strain. However the promoter of *sopB* located in SPI-5 remained active but its activity was marginally lower than in the wild-type strain (Figure 3B). The regulation of many T3SS genes often require multiple signals for maximal expression and clearly other signals remain in the Δ*mreC* depletion mutant which drive expression of the SopB in SPI-5.

Expression of SPI-2 T3SS genes were monitored using a transcriptional reporter for the SPI-2 gene *ssaG*, whose promoter was cloned upstream of the *luxCDABE* luciferase operon in the plasmid pMK1-*lux* [52]. The construct was transformed into wild-type SL1344 and Δ*mreC*, and the luminescence and OD600 measured during growth in SPI-2 inducing conditions (Figure 3B). The *ssaG* promoter remains active in the Δ*mreC* mutant although expression appears to be delayed, and is marginally less than in WT. This evidence supports the western blot data with αSseB and suggests that in contrast to the SPI-1 T3SS, the SPI-2 T3SS remains functional in the absence of the cytoskeleton.

### Function of the RcsC Two-Component System in Regulation of SPI-1 T3S and Motility in Δ*mreC*

Two-component regulatory systems are vital in sensing environmental and cell surface signals, enabling bacteria to rapidly adapt to ever changing conditions [53], [54]. These signals are detected by histidine protein sensor kinases, which subsequently transfer phosphate groups to an aspartate residue in the response regulator proteins, thus modulating their regulatory activities. The environmental signals are thus transmitted by a phosphorelay system to regulate gene expression.

**Figure 5:**
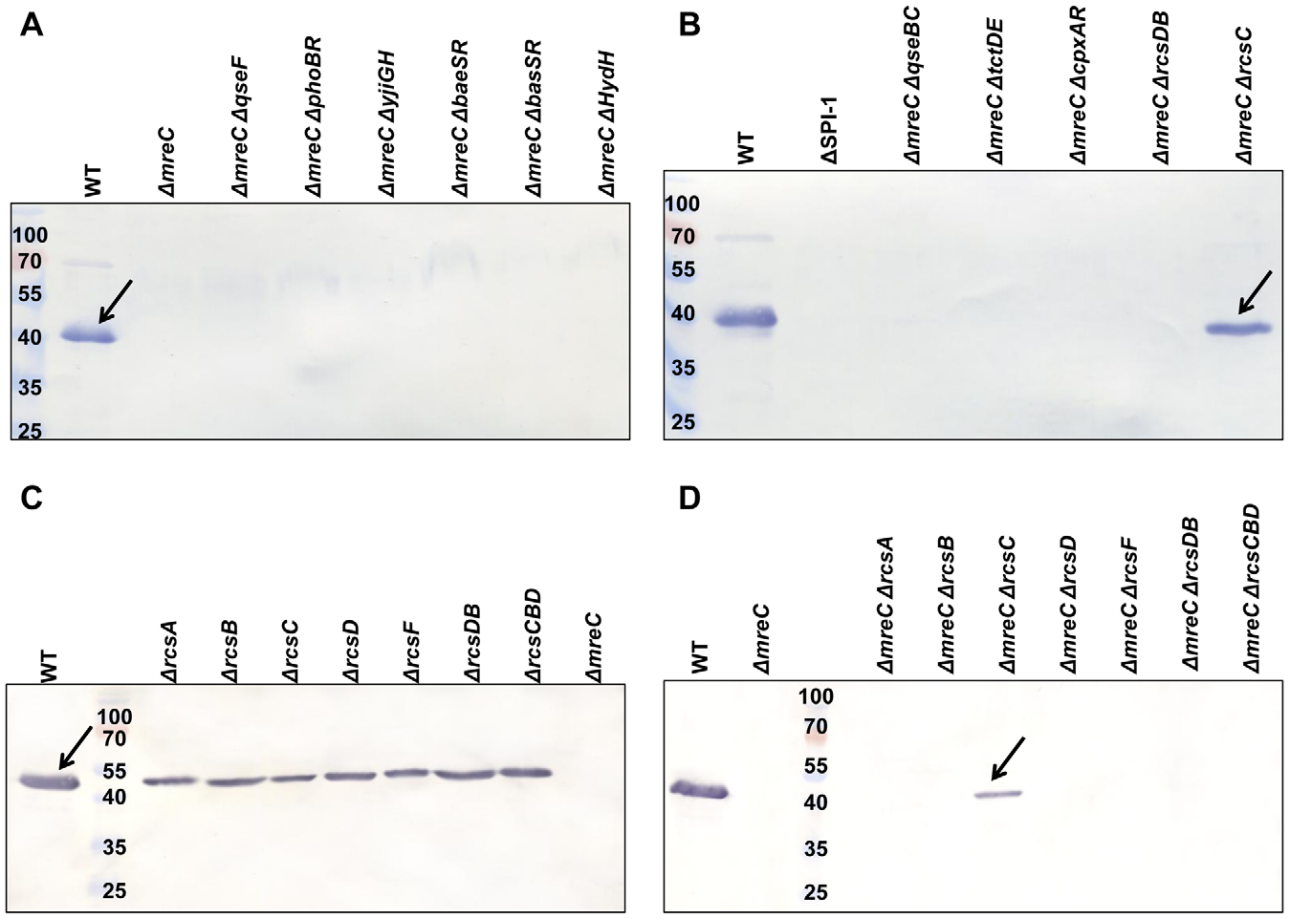
Western blotting screen of Δ*mreC* two-component system double mutants for recovery of SPI-1 T3S. Panels A and B show western blots of total protein samples obtained from SL1344 WT, ΔSPI-1, Δ*mreC*, Δ*mreC* Δ*qseF,* Δ*mreC* Δ*phoBR*, Δ*mreC* Δ*yjiGH*, Δ*mreC* Δ*baeSR*, Δ*mreC* Δ*basSR*, Δ*mreC* Δ*hydH*, Δ*mreC* Δ*qseBC*, Δ*mreC* Δ*tctDE,* Δ*mreC* Δ*cpxAR,* Δ*mreC* Δ*rcsDB, and* Δ*mreC* Δ*rcsC* strains with αSipC antibody. Panels C and D show western blot of total protein samples obtained from SL1344 WT, *ΔrcsA, ΔrcsB, ΔrcsC*, *ΔrcsD, ΔrcsF, ΔrcsDB, ΔrcsCBD* and *ΔmreC* strains along with the *ΔmreC ΔrcsA, ΔmreC ΔrcsB, ΔmreC ΔrcsC, ΔmreC ΔrcsD, ΔmreC ΔrcsF, ΔmreC ΔrcsDB*, and *ΔmreC ΔrcsCBD* double mutants with αSipC antibody. SipC is indicated at approximately 43kDa.

**Figure 6:**
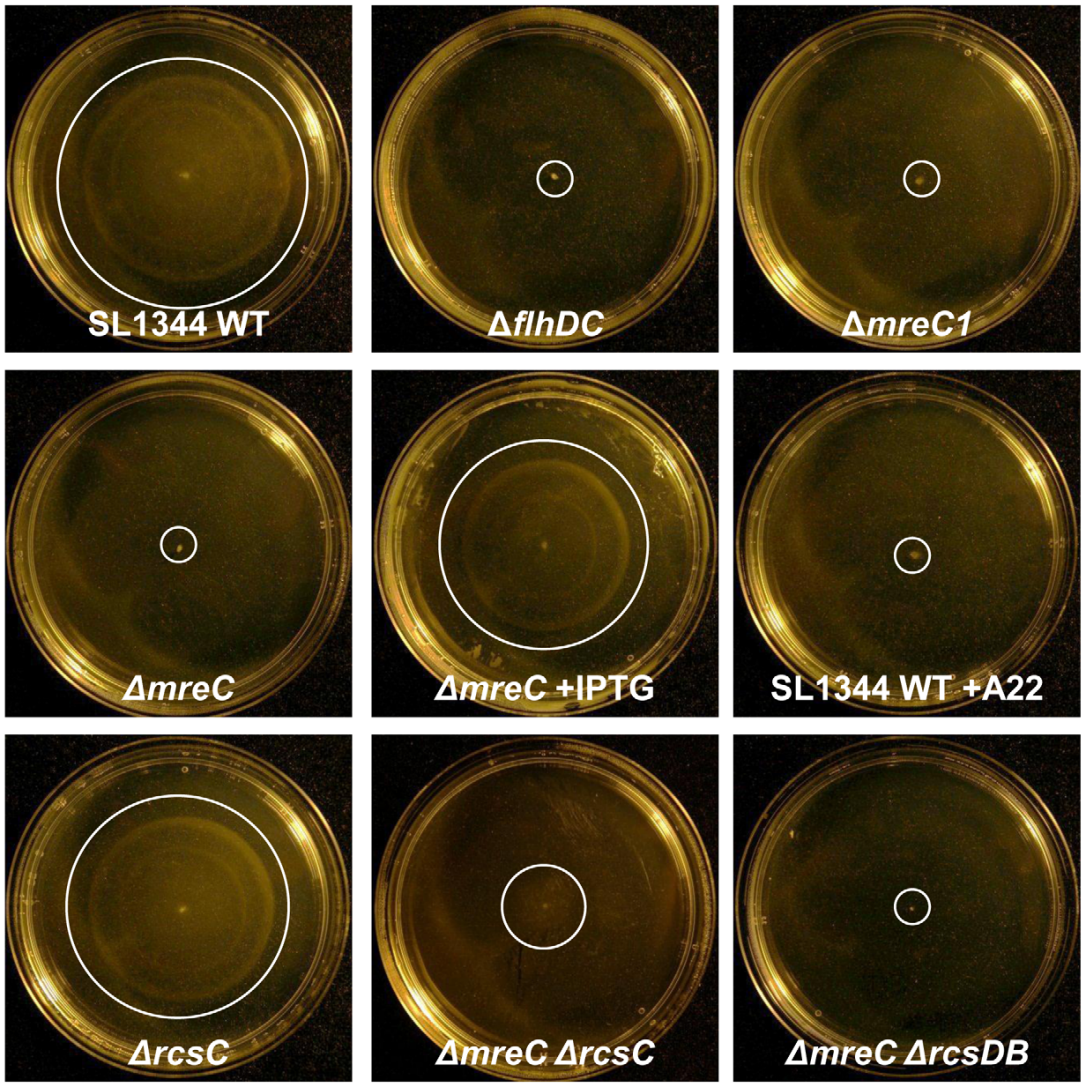
Motility of *Salmonella* mutant cells. Representative images showing the motility of SL1344 WT, Δ*flhDC* Δ*mreC1*, Δ*mreC*, Δ*mreC* plus IPTG, Δ*rcsC*, Δ*mreC* Δ*rcsC*, Δ*mreC* Δ*rcsDB,* and SL1344 WT plus A22 cells grown on motility agar at 37°C. White circles highlight the limits of motility on the agar plates.

In order to identify putative regulators of the Δ*mreC* observed phenotypes, we have constructed knockout mutations in a range of two-component systems. As an initial screen, a panel of nine separate two-component system mutant strains were constructed as double mutants with Δ*mreC*. One two-component system sensor kinase mutation Δ*rcsC* resulted in recovery of SPI-1 effector expression in the Δ*mreC* background as judged by western blotting using αSipC sera (Figure 5 panels A and B). Interestingly the amount of SipC protein expressed and secreted from the cell was less than the wild-type suggesting there are additional repressors continuing to operate (Figure 5 panels A and B and Figure S5). Furthermore, disruption of *rcsC* also significantly de-repressed motility (Figure 6 and Figure S6) in a Δ*mreC* mutant similar to SPI-1 expression, again suggesting there are additional repressors involved. Expression of the RcsC protein *in trans* was able to restore the phenotype of Δ*mreC* Δ*rcsC* back to the equivalent of a Δ*mreC* strain, with respect to repressing SPI-1 type 3 secretion and motility. These complementation studies provide further evidence supporting the regulatory role of RcsC in the Δ*mreC* phenotypes (Figure S7).

Rcs is a highly complex multi-component phosphorelay system and was originally identified in regulating genes involved in capsule synthesis in *Escherichia coli* [55], [56]. The RcsC sensor kinase phosphorylates RcsD, which subsequently phopshorylates the DNA binding response regulator RcsB. The unstable RcsA protein and additional auxillary proteins can also interact and regulate RcsB. The Rcs system is involved in down-regulating the expression of flagella, SPI1-T3S and increasing biofilm formation [57].

We therefore also constructed Δ*mreC* Δ*rcsB*, Δ*mreC* Δ*rcsD*, Δ*mreC* Δ*rcsDB* and Δ*mreC* Δ*rcsCBD* mutants, which however did not restore either SPI-T3S or motility (Figures 5, 6, and S6). We propose that in the absence of RcsC signalling, phosphorylated levels of RcsB are depleted enabling de-repression of FlhDC and motility. The presence of RcsDB appears essential for restoring motility in the absence of RcsC [55]. The functionality of SPI-1 T3SS in the Δ*mreC* Δ*rcsC and* Δ*mreC* Δ*rcsDB* mutants were assessed in a TER assay, which revealed partial restoration of tight junction disruption in the Δ*mreC* Δ*rcsC* mutant, but not in the Δ*mreC* Δ*rcsDB* (Figure S8).

It has been suggested that the outer membrane protein RcsF may perceive some of the environmental signals necessary to activate the Rcs phosphorelay system. To investigate this we constructed a Δ*mreC* Δ*rcsF* mutant which failed to restore motility or SPI-1 T3S and appeared phenotypically identical to Δ*mreC* (Figure 5, S6). This would suggest that RcsF is not involved in the observed Δ*mreC* phenotypes. Furthermore as the auxillary protein RcsA can interact and regulate RcsB, we therefore disrupted the *rcsA* gene in Δ*mreC* and which also resulted in no impact on the observed phenotypes (Figure 5, S6).

In summary, we propose that RscC is sensing cell surface perturbations [58] in Δ*mreC*, resulting from a disrupted cytoskeleton, and subsequently down-regulating the expression of SPI-1 T3S and motility. This signalling appears to be independent of both RcsF and RcsA.

### Chemical Genetic Inactivation of the Essential MreB Protein

A cell permeable compound named A22 [*S*-(3,4-Dichlorobenzyl) isothiourea] has been demonstrated to perturb MreB function [59]. As an alternative approach to genetically disrupting the essential gene *mreB*, we exposed wild-type *Salmonella* cultures to A22 and observed a morphological change from rod to spherical-shaped cells. In addition we phenotypically screened and tested A22-treated cells for motility and T3S. The A22-treated cells were phenotypically identical to Δ*mreC* with respect to cell shape, motility, SPI-1 T3S, and also SPI-2 T3S (data not shown). The effects of A22 were completely reversible following its removal (data not shown). Thus the chemical genetic inactivation of MreB, independently corroborates the phenotypic observations made with Δ*mreC.*

### The *Salmonella mre* Operon Plays an Important Role in Colonization during *in vivo* Infection

**Figure 7:**
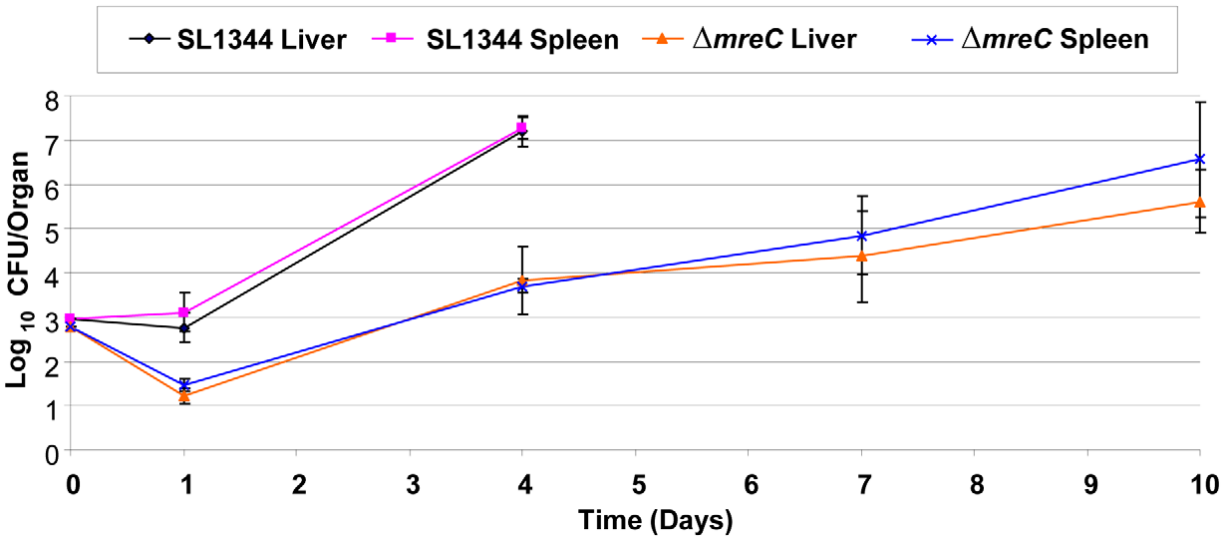
Contribution of Δ*mreC* to *in vivo* colonization. *In vivo* growth kinetics of WT SL1344 and Δ*mreC* in livers and spleens of C57BL/6 mice inoculated intravenously with 10^3^ colony forming units. Viable bacterial counts in the spleen and liver were performed at days 1, 4, 7 and 10, and expressed as mean log^10^ viable count +/− standard deviation.

The Δ*mreC* defect clearly has an impact on the expression of important virulence determinants of *Salmonella in vitro*. We therefore wished to investigate the contribution of the bacterial cytoskeleton on the virulence of *Salmonella in vivo* using the mouse model. We observed that the SPI-1 T3SS in Δ*mreC* is completely down-regulated, and as this virulence system is important for infection through the oral route of inoculation the strain would be attenuated. We therefore explored the colonization of Δ*mreC* using the intravenous route allowing us to examine the impact of the host on the further down-stream stages of infection. Groups of 5 female C57/BL6 mice were inoculated intravenously with *circa* 103 colony forming units of either control SL1344 or Δ*mreC*. The times taken for clinical symptoms to appear were determined. Viable bacterial numbers in the spleen and liver for SL1344 were determined at days 1 and 4, and Δ*mreC* at days 1, 4, 7, and 10. The *in vivo* bacterial net growth curves vividly demonstrate two clear phenotypic effects upon the growth of Δ*mreC* compared to the wild-type. Firstly, there is a greater initial kill of Δ*mreC*, and this is secondly followed by a slower net growth rate. However, in spite of the reduced growth rate of Δ*mreC*, the bacterial numbers steadily increase over 6 days. This eventually causes the onset of clinical symptoms necessitating termination of the experiment at day 10 (Figure 7). During these stages *Salmonella* infect and multiply within macrophages and the SPI-2 T3SS is essential for survival. Thus providing further evidence to support the presence of a functional SPI-2 T3SS in Δ*mreC*. Collectively, these observations imply the *mreC* defect reduces the virulence of the strain, but does not completely abrogate its ability to multiply and cause disease systemically *in vivo*.

### Morphology *in vivo*

**Figure 8:**
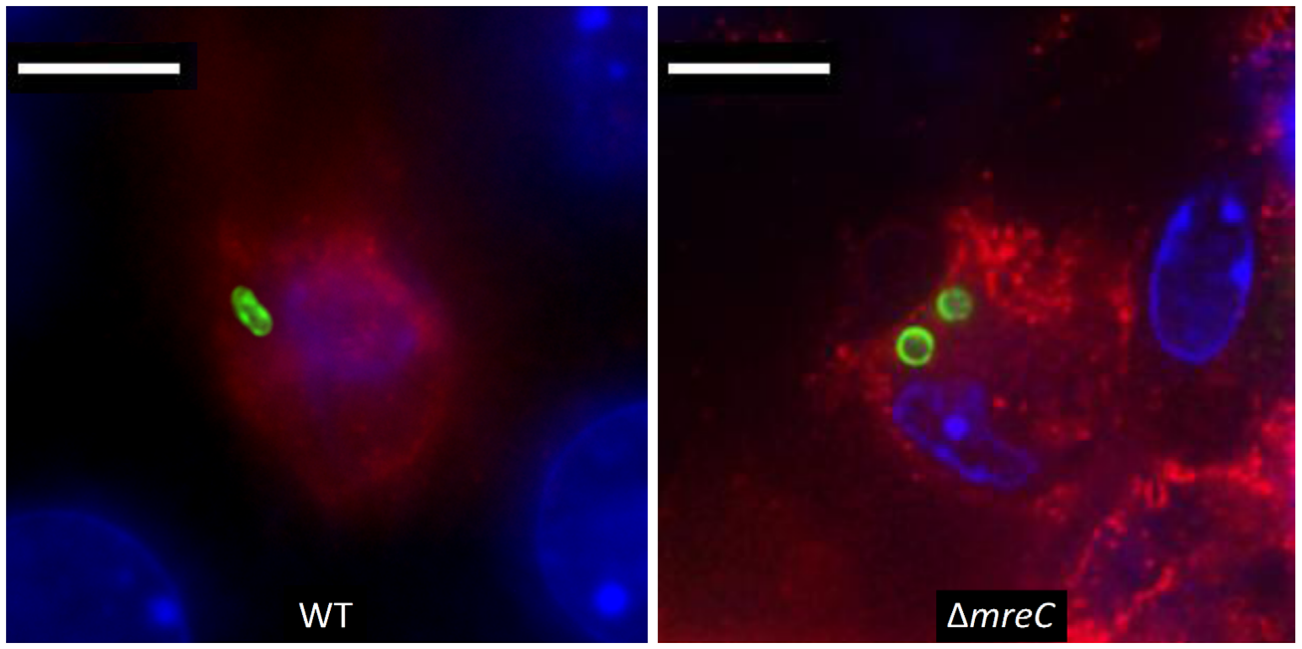
Morphology of Δ*mreC* in host tissues. Representative fluorescence micrograph of *Salmonella* SL1344 WT and Δ*mreC* within a phagocyte in infected livers of C57BL/6 mice at 72 h p.i. CD18+ expressing cells (red), *Salmonella* Δ*mr*eC (green), nucleic acid is indicated by DAPI (blue). Scale bar, 5 µm.

Strains recovered from *in vivo* passage were tested for changes in morphology, motility and T3S, and were found to be identical to the input strain. Furthermore the *in vivo* morphology of the strain within livers and spleens was determined by immunofluorescence microscopy. Sections of livers and spleens were taken and stained as described in the materials and methods. Figure 8 demonstrates the *Salmonella* Δ*mreC* mutant strain retains the round morphology *in vivo* compared to the rod shaped wild-type control. Collectively these data suggests that the mutation has remained stable during the *in vivo* passage for the virulence phenotypes tested.

### Role of the Cytoskeleton in the Assembly, Regulation and Function of SPI-1 T3SS and Flagella

**Figure 9:**
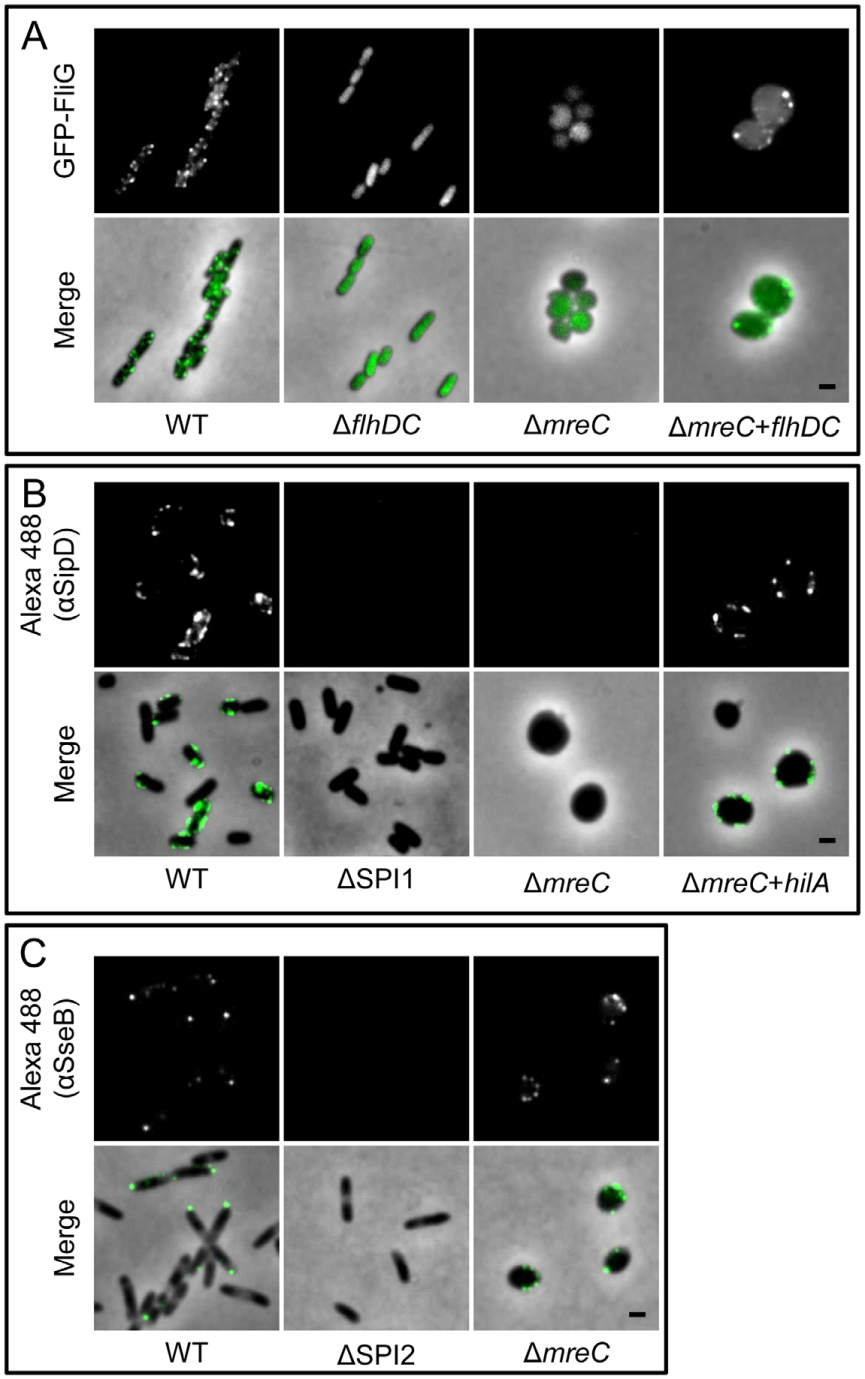
Localization of flagella and Type 3 secretion systems. Panel A shows representative images of *Salmonella* SL1344 WT, Δ*flhDC*, Δ*mreC*, and flagella-complemented Δ*mreC* pTET*flhDC* cells. Panel B shows representative images of *Salmonella* SL1344 WT, ΔSPI1, Δ*mreC*, and SPI-1 complemented Δ*mreC* pBAD*hilA* cells. Panel C shows representative images of *Salmonella* SL1344 WT, ΔSPI2, and Δ*mreC*. Fluorescence images of (A) GFP-FliG, (B) Alexa488-αSipD or (C) Alexa488-αSseB (top panels) and phase merged images (bottom panels) are shown in each panel. Scale bar representing 1 µm is indicated in the bottom right panel.

The regulation and assembly of SPI-1 T3SS and flagella are complex. When the bacterial cytoskeleton is disrupted both the SPI-1 T3SS and flagella expression are down-regulated. A hypothesis is that the integrity of the cytoskeleton is essential for the correct assembly of these complex macromolecular structures and in its absence the SPI-1 and flagella gene expression are down-regulated to conserve resources. Alternatively, in the absence of a functional cytoskeleton the bacterial cell is stressed and shuts down the expression of energetically expensive “non-essential” machinery. To test these ideas we wished to force on the expression of SPI-1 T3S and flagella genes, and examine whether these systems are correctly assembled and functional. We therefore expressed *in trans* from heterologous inducible promoters either the flagella master regulator FlhDC or the SPI-1 T3S regulator HilA in a panel of strains including Δ*mreC.* Strikingly, expression of FlhDC restored both the expression and assembly of flagella on the cell surface as determined by fluorescence microscopy (Figure 9A) and motility assays (data not shown) in Δ*mreC.* Furthermore, expression of HilA *in trans* up-regulated expression of the SPI-T3SS and its assembly on the cell surface as determined immunofluorescence microscopy (Figure 9B) western blotting with αSipB antibody (Figure S9) or functionally by TER measurements (Figure 4). In contrast to SPI-1 T3SS and flagella, the expression of the SPI-2 T3SS was not turned off in the Δ*mreC* mutant as shown in (Figure 9C). Interestingly, in WT cells the SPI-1 T3S apparatus and flagella appear to be present in around six to eight copies mainly along the long axis of the cell. In marked contrast the SPI-2 apparatus is typically present in one or two copies located at the poles of the bacterial cell [42], whereas their localisation appears less clear in the Δ*mreC* mutant, possibly due to perturbations in the cell envelope and the indistinct cell polarity in these cells caused by disruption of the cytoskeleton. The complementation of the functional assembly of SPI-1 T3SS was also confirmed using TER assays, where the levels of decrease in resistance after infection with Δ*mreC* strain reverted to that of the parent strain upon induction of the transcriptional regulator *hilA* (Figure 9B and S9), or complementation of the Δ*mreC* mutation (Figure 4). Taken together the data support the notion that the cytoskeleton is not required for the correct assembly of these virulence factors but essential for their expression.

## Discussion

Bacterial cells possess dynamic cytoskeletons composed of diverse classes of self-assembling polymeric proteins. Some of these proteins resemble eukaryotic actin, tubulin, and intermediate filaments both structurally and functionally [5], [7], [11], [12]. The bacterial tubulin FtsZ plays a key role in cell division. Bacterial actins provide vital functions in maintaining cell morphology, segregating DNA, and positioning bacterial organelles. It has recently been demonstrated in *Helicobacter pylori*, that MreB is essential not for cell shape but for maintenance of the full enzymatic activity of urease, an essential virulence factor [60]. Furthermore the MreB cytoskeleton is also essential for the polar localisation of pili in *Pseudomonas aeruginosa* [61].

Using a variety of approaches we have demonstrated the importance of the bacterial cytoskeleton in the pathogenicity of *Salmonella*. MreC and MreD form a complex in the cytoplasmic membrane, which subsequently interacts with MreB. The *mreB* gene appears to be essential in many organisms including as we discovered in *Salmonella*. Viable *mreB* mutants often contain compensatory changes in other genes e.g. *ftsZ* which compensate for the lethality of the *mreB* lesion [37]. As an alternative strategy to investigate the function of the bacterial cytoskeleton and avoid these deleterious effects, we carefully constructed depletion mutants of *mreC* in strains harbouring a single-copy plasmid expressing the MreB operon from the *lac* promoter. In addition we confirmed the phenotypic effects of the *mreC* genetic lesion by disrupting the functions of MreB using a chemical genetics approach and inactivating MreB with A22.

Removal of the gratuitous inducer IPTG from the growth medium of the Δ*mreC* depletion mutant resulted in cells changing from rod to a spherical shaped morphology. Using fluorescence microscopy MreB was observed to be no longer distributed in a helical fashion throughout the cell but rather diffusely throughout the cytoplasm (data not shown). Presumably MreB polymers are no longer able to contact the cytoplasmic membrane via MreD attachment sites resulting in mis-assembly of the entire cytoskeleton. In growing cells, this disruption of the cytoskeleton leads to loss of the rod-shape.

We next examined the motility of Δ*mreC* depletion strain to assess the functionality of flagella. The strains were non-motile and western blotting revealed absence of the flagellin filament subunit proteins FliC and FljB in both secreted and also cytoplasmic protein fractions, suggesting expression of these alternative subunits had been switched off. Flagella gene expression is complex and involves a regulatory hierarchy of Class I, Class II, and Class III genes [38]. The class I *flhDC* operon is the master regulator, and FlhDC complex is required for transcriptional activation of the class II genes including the specialized flagellar sigma factor FliA. FliA alone or with FlhDC complex, activates expression of the class III operon genes encoding motor proteins, hook-associated proteins, the filament protein, and chemotaxis proteins [39], [40]. Expression of the FlhDC complex was reduced but still appeared comparable between the wild-type and the Δ*mreC* suggesting changes in the promoter activity of *flhDC alone* are not responsible for the observed phenotype. Class II gene expression was significantly reduced. Expression of the Class III gene *fliC* was completely down-regulated confirming the western blot observations. Hence these independent observations are in accordance with the Δ*mreC* motility data. Thus in the absence of the cytoskeleton expression of class II and class III flagella genes appears to be down-regulated.

Expression of the SPI-1 T3S system is essential for invasion of intestinal epithelial cells and the SPI-2 T3SS plays a central role in survival within the hostile environment of a macrophage [43]. Western blotting revealed the SPI-1 T3S structural protein PrgH and the effectors SipA and SipC were no longer expressed or secreted in the Δ*mreC* depletion mutant. The phenoptype was fully complementable by the addition of IPTG. Several environmental signals and transcriptional factors modulate expression of the SPI-1 and SPI-2 T3SS [43], [45], [62]. We wished to understand the mechanistic basis by which expression of the SPI1-T3SS is down-regulated. Within SPI-1 there are key transcriptional activators which regulate expression of SPI-1 genes: HilC, HilD, HilA, and InvF. Using promoter-luciferase transcriptional reporter assays it was revealed that the SPI-1 transcription factor gene promoters for *hilA*, *hilC*, *hilD*, and *invF* were completely inactive in Δ*mreC,* in marked contrast to the control wild-type strain. Surprisingly, the promoter of *sopB* located outside of SPI-1 in SPI-5 remained active but its activity was marginally lower than in the wild-type strain. The regulation of many T3SS genes often require the input of multiple signals for maximal expression and clearly other signals remain in the Δ*mreC* depletion mutant which drive expression of the SopB in SPI-5. It therefore appears that the SPI-1 T3SS is completely down-regulated in the absence of an cytoskeleton by an unidentified regulatory factor. In contrast, the SPI-2 T3SS remains functional as evidenced by western blotting with SseB antibody and promoter-reporter assays. This is further corroborated with the *in vivo* evidence that following systemic inoculation, Δ*mreC* is able to survive and multiply within the host. This takes place within the hostile environment of the macrophage where SPI-2 T3S is essential for biogenesis of the *Salmonella* containing vacuole and survival [43], [63], [64].

We wished to gain further insights into the mechanistic basis of the down-regulation of both SPI- T3SS and motility in Δ*mreC*. Two-component systems play an essential role in sensing and responding to environmental and cell surface signals [54]. To investigate if two-component systems contribute to the regulation of the Δ*mreC* phenotypes, we constructed a panel of separate two-component system mutant strains in an *ΔmreC* background. The double mutants were screened for recovery of motility and expression of the SPI-1 T3SS. A mutation in the *rcsC* sensor kinase gene resulted in significant but not complete recovery of both motility and expression of the SPI-1 T3SS.

The Rcs phosphorelay system regulates a broad range of genes from capsule synthesis in *E. coli* to increasing biofilm formation [58]. RcsC also plays an important role in repressing expression of flagella and SPI-1 T3SS in *Salmonella* Typhi [57]. The RcsC sensor kinase normally phosphorylates RcsD, which subsequently phosphorylates the DNA binding response regulator RcsB. However, in Δ*mreC* Δ*rcsDB* and Δ*mreC* Δ*rcsCBD* there was no restoration of either motility or expression of the SPI-1 T3SS suggesting that RcsC signals repression and requires the presence of *rcsDB* to mediate this effect. We propose that in Δ*mreC*, the sensor kinase RscC detects cell surface perturbations and down-regulates expression of flagella and the SPI-1 T3S apparatus [58]. This signalling is independent of both the outer membrane lipoprotein RcsF sensor and the auxilliary regulatory protein RcsA.

There are a number of explanations to provide a bacterial rational for this shutdown in expression. In the absence of a functional cytoskeleton the flagella and SPI-1 T3SS are either not being correctly assembled, triggering a feedback loop to repress expression, or alternatively are down-regulated to prevent the cell from wasting valuable resources under these conditions. To test the assembly idea, we forced on the expression of flagella and SPI-1 T3SS genes by expressing the regulators *flhDC* or *hilA in trans* in Δ*mreC.* Using independent methods we observed the correct assembly and function of these macromolecular machines suggesting the cytoskeleton is not essential for functionality. The cytoskeleton could also have a role in sensing cellular stress, as has recently been suggested by Chiu and colleagues [65]. They propose that the integrity of the cytoskeleton may be exploited by the cell to monitor oxidative stress and physiological status. If the cytoskeleton disintegrates in the absence of MreC, this may be sensed by the cell leading to a shut-down of the SPI-1 T3S apparatus and down-regulation of flagella protein expression. We have provided mechanistic insights into the regulation of motility and SPI-1 T3S in Δ*mreC*. We have identified the two-component system sensor RcsC as an important regulator controlling expression of these systems, presumably as a consequence of sensing membrane perturbations brought about by the disruption of the cytoskeleton [58].

With a non-functional SPI-1 T3SS, we would expect the Δ*mreC* would be attenuated in mice when administered by the oral route as it is unable to invade intestinal epithelial cells by the SPI-1 T3SS. We therefore explored the colonization of Δ*mreC in vivo* using the intravenous route of inoculation [66]. This provides an opportunity to examine the impact of Δ*mreC* on the down-stream stages of infection. *Salmonella* infect and multiply within macrophages during the systemic stages of infection. Survival within the hostile environment of the macrophage would require a functional SPI-2 T3SS in the *Salmonella*-containing vacuole to remodel the host cell environment and survive attack from reactive oxygen free radicals [64], [67], [68]. By examining the *in vivo* net bacterial growth curves within livers and spleens two clear phenotypic effects were revealed with Δ*mreC* compared to the wild-type. Greater initial killing of Δ*mreC* is followed by a slower net growth rate with the bacterial numbers steadily increasing over six days. Clinical symptoms begin to appear and by day ten these symptoms necessitate termination of the experiment. The phenotypic data clearly imply the Δ*mreC* defect reduces the colonization of *Salmonella*, but does not completely abrogate its ability to multiply and cause disease systemically *in vivo*. This would suggest that the second T3S in *Salmonella* encoded on SPI-2 remains sufficiently functional to permit growth in the absence of the cytoskeleton.

In the absence of an intact cytoskeleton in Δ*mreC* the expression of the SPI-1 T3SS and flagella are clearly down-regulated. Strikingly however, the SPI-2 T3SS appears to remain functional contributing to the virulence of the Δ*mreC* strain observed *in vivo*. A possible explanation could be that the regulation of the SPI-2 T3SS is co-ordinated independently of the integrity of the cytoskeleton in contrast to flagella and SPI-1 T3SS. Collectively these data highlight the importance of the bacterial cytoskeleton in the ability of *Salmonella* to cause disease, and may provide opportunities for the development of new antimicrobials to target the cytoskeleton.

## Supporting Information

Figure S1 **Expression of MreC in complemented Δ** ***mreC*** **cells.** Western blot of total protein samples from SL1344 WT, Δ*mreC1*, Δ*mreC*, and Δ*mreC* plus 100 µM IPTG cells using αMreC antibody. MreC is indicated at approximately 38kDa and is distinguishable from background bands.
(TIF)

Figure S2 **Growth curve of** ***Salmonella*** **mutant cells.** Log phase growth of SL1344 WT, Δ*mreC1*, Δ*mreC*, Δ*mreC* plus 100 µM IPTG, and A22 treated SL1344 WT cells. Strains were grown in LB media at 37°C.
(TIF)

Figure S3 **Motility of** ***Salmonella*** **Δ** ***mre*** **mutant cells.** Motility of SL1344 WT, Δ*flhDC*, Δ*mreC1*, Δ*mreC*, Δ*mreC* plus 100 µM IPTG, and A22 treated SL1344 WT shown as a percentage of the wild type. Strains were grown on motility agar at 37°C. Experiments were repeated at least three times and error bars indicate SD. ^*^ Indicates statistical difference from WT (p<0.05).
(TIF)

Figure S4 **Translocation of SipB SPI-1 effector protein into Caco-2 cells.** Western blot of host cytosol fractions with αSipB antibody following infection of cells with Salmonella SL1344 WT, ΔSPI-1, Δ*mreC1*, Δ*mreC* (+/− IPTG) mutants. SipB is indicated at approximately 65kDa.
(TIF)

Figure S5 **Secretion of SPI-1 effector protein SipC in Δ** ***rcs*** **C mutant cells.** Western blot of secreted protein samples from SL1344 WT, Δ*mreC*, ΔSPI-1, ΔSPI-2, Δ*rcsC*, and Δ*mreC* Δ*rcsC* cells using αSipC antibody. SipC is indicated at approximately 43kDa.
(TIF)

Figure S6 **Motility of** ***Salmonella*** **Δ** ***rcs*** **mutant cells.** Motility of SL1344 WT, *ΔmreC, ΔflhDC, ΔrcsA, ΔrcsB, ΔrcsC, ΔrcsD, ΔrcsF, ΔrcsDB, ΔrcsCBD, ΔmreC ΔrcsA, ΔmreC ΔrcsB, ΔmreC ΔrcsC, ΔmreC ΔrcsD, ΔmreC ΔrcsF, ΔmreC ΔrcsDB,* and *ΔmreC ΔrcsCBD* cells shown as a percentage of the wild type. Experiments were repeated at least three times and error bars indicate SD. Strains were grown on motility agar at 37°C.
(TIF)

Figure S7 **Effect of** ***rcsC*** **expression on SipC production and motility.** Panels A and B show western blots from SL1344 WT, mreC, and SPI-1 control strains, and SL1344 WT pBAD*rcsC*, mreC pBADrcsC, *rcsC* pBAD*rcsC*, and *mreC rcsC* pBAD*rcsC* strains (+/− arabinose) with αSipC antibody. SipC is indicated at approximately 43kDa. Panel C shows motility of SL1344 WT, mreC, SL1344 WT pBAD*rcsC*, mreC pBADrcsC, *rcsC* pBAD*rcsC*, and *mreC rcsC* pBAD*rcsC* strains (+/− arabinose) shown as a percentage of the wild type. Experiments were repeated at least three times and error bars indicate standard deviation.
(TIF)

Figure S8 **Percentage change in transepithelial resistance of differentiated Caco-2 cells after 4hr infection with Δ** ***rcs*** **mutant strains.** TER of polarised Caco-2 monolayers exposed to *Salmonella* strains at an MOI of 20. TER change is expressed as a percentage alteration at 4hr compared to the initial value at time zero. Error bars indicate the standard deviations derived from at least three independent experiments. ^*^ Indicates statistical difference from WT (p<0.05).
(TIF)

Figure S9 **Complementation of** ***Salmonella*** **Pathogenicity Island SPI-1 in Δ** ***mreC*** **mutant.** Expression of SPI-1 proteins in WT SL1344, ΔSPI-1, and Δ*mreC* mutants, and complemented Δ*mreC* pBAD*hilA* strain during SPI-1 inducing conditions as revealed by western blotting with polyclonal αSipB antibody. SipB is indicated at approximately 63kDa, and a breakdown product is evident.
(TIF)

## References

[1] Pang T, Levine MM, Ivanoff B, Wain J, Finlay BB (1998). Typhoid fever-important issues still remain.. Trends Microbiol.

[2] Lilic M, Galkin VE, Orlova A, VanLoock MS, Egelman EH (2003). Salmonella SipA polymerizes actin by stapling filaments with nonglobular protein arms.. Science.

[3] Piddock LJ (2006). Multidrug-resistance efflux pumps - not just for resistance.. Nat Rev Microbiol.

[4] Mirza SH, Beeching NJ, Hart CA (1996). Multi-drug resistant typhoid: a global problem.. J Med Microbiol.

[5] Jones LJ, Carballido-Lopez R, Errington J (2001). Control of cell shape in bacteria: helical, actin-like filaments in Bacillus subtilis.. Cell.

[6] Bi E, Lutkenhaus J (1991). FtsZ ring structure associated with division in Escherichia coli.. Nature.

[7] Lowe J, Amos LA (1998). Crystal structure of the bacterial cell-division protein FtsZ.. Nature.

[8] Lutkenhaus J, Addinall SG (1997). Bacterial cell division and the Z ring.. Annu Rev Biochem.

[9] Ausmees N, Kuhn JR, Jacobs-Wagner C (2003). The bacterial cytoskeleton: An intermediate filament-like function.. Cell.

[10] Carballido-Lopez R, Errington J (2003). A dynamic bacterial cytoskeleton.. Trends Cell Biol.

[11] van den Ent F, Amos L, Löwe J (2001). Bacterial ancestry of actin and tubulin.. Curr Opin Microbiol.

[12] van den Ent F, Amos LA, Lowe J (2001). Prokaryotic origin of the actin cytoskeleton.. Nature.

[13] Kruse T, Bork-Jensen J, Gerdes K (2005). The morphogenetic MreBCD proteins of Escherichia coli form an essential membrane-bound complex.. Mol Microbiol.

[14] Kruse T, Moller-Jensen J, Lobner-Olesen A, Gerdes K (2003). Dysfunctional MreB inhibits chromosome segregation in Escherichia coli.. EMBO J.

[15] Formstone A, Errington J (2005). A magnesium-dependent mreB null mutant: implications for the role of mreB in Bacillus subtilis.. Mol Microbiol.

[16] Gitai Z, Dye N, Shapiro L (2004). An actin-like gene can determine cell polarity in bacteria.. Proc Natl Acad Sci U S A.

[17] Figge RM, Divakaruni AV, Gober JW (2004). MreB, the cell shape-determining bacterial actin homologue, co-ordinates cell wall morphogenesis in Caulobacter crescentus.. Mol Microbiol.

[18] Wachi M, Doi M, Okada Y, Matsuhashi M (1989). New Mre Genes Mrec and Mred, Responsible for Formation of the Rod Shape of Escherichia-Coli-Cells.. J Bacteriol.

[19] Divakaruni AV, Loo RRO, Xie Y, Loo JA, Gober JW (2005). The cell-shape protein MreC interacts with extracytoplasmic proteins including cell wall assembly complexes in Caulobacter crescentus.. Proc Natl Acad Sci U S A.

[20] Vats P, Shih YL, Rothfield L (2009). Assembly of the MreB-associated cytoskeletal ring of Escherichia coli.. Mol Microbiol.

[21] Slovak PM, Porter SL, Armitage JP (2006). Differential localization of Mre proteins with PBP2 in Rhodobacter sphaeroides.. J Bacteriol.

[22] Gitai Z, Dye NA, Reisenauer A, Wachi M, Shapiro L (2005). MreB actin-mediated segregation of a specific region of a bacterial chromosome.. Cell.

[23] Madabhushi R, Marians KJ (2009). Actin homolog MreB affects chromosome segregation by regulating topoisomerase IV in Escherichia coli.. Mol Cell.

[24] Kruse T, Blagoev B, Lobner-Olesen A, Wachi M, Sasaki K (2006). Actin homolog MreB and RNA polymerase interact and are both required for chromosome segregation in Escherichia coli.. Genes Dev.

[25] Shiomi D, Sakai M, Niki H (2008). Determination of bacterial rod shape by a novel cytoskeletal membrane protein.. EMBO J.

[26] Bendezu FO, Hale CA, Bernhardt TG, de Boer PA (2009). RodZ (YfgA) is required for proper assembly of the MreB actin cytoskeleton and cell shape in E. coli.. EMBO J.

[27] Alyahya SA, Alexander R, Costa T, Henriques AO, Emonet T (2009). RodZ, a component of the bacterial core morphogenic apparatus.. Proc Natl Acad Sci U S A.

[28] Ghosh AS, Young KD (2005). Helical Disposition of Proteins and Lipopolysaccharide in the Outer Membrane of Escherichia coli.. J Bacteriol.

[29] Taghbalout A, Rothfield L (2008). RNaseE and RNA helicase B play central roles in the cytoskeletal organization of the RNA degradosome.. J Biol Chem.

[30] Ehrbar K, Mirold S, Friebel A, Stender S, Hardt WD (2002). Characterization of effector proteins translocated via the SPI1 type III secretion system of Salmonella typhimurium.. Int J Med Microbiol.

[31] Datsenko KA, Wanner BL (2000). One-step inactivation of chromosomal genes in Escherichia coli K-12 using PCR products.. Proc Natl Acad Sci U S A.

[32] Morimoto YV, Nakamura S, Kami-ike N, Namba K, Minamino T (2010). Charged residues in the cytoplasmic loop of MotA are required for stator assembly into the bacterial flagellar motor.. Mol Microbiol.

[33] Karlinsey JE, Tanaka S, Bettenworth V, Yamaguchi S, Boos W (2000). Completion of the hook-basal body complex of the Salmonella typhimurium flagellum is coupled to FlgM secretion and fliC transcription.. Mol Microbiol.

[34] Dean P, Kenny B (2004). Intestinal barrier dysfunction by enteropathogenic Escherichia coli is mediated by two effector molecules and a bacterial surface protein.. Mol Microbiol.

[35] McClelland M, Sanderson KE, Spieth J, Clifton SW, Latreille P (2001). Complete genome sequence of Salmonella enterica serovar Typhimurium LT2.. Nature.

[36] Shih YL, Le T, Rothfield L (2003). Division site selection in Escherichia coli involves dynamic redistribution of Min proteins within coiled structures that extend between the two cell poles.. Proc Natl Acad Sci USA.

[37] Bendezu FO, de Boer PAJ (2008). Conditional Lethality, Division Defects, Membrane Involution, and Endocytosis in mre and mrd Shape Mutants of Escherichia coli.. J Bacteriol.

[38] Macnab RM (2003). How bacteria assemble flagella.. Ann Rev Microbiol.

[39] Komeda Y (1982). Fusions of flagellar operons to lactose genes on a mu lac bacteriophage.. J Bacteriol.

[40] Kutsukake K, Ohya Y, Iino T (1990). Transcriptional analysis of the flagellar regulon of Salmonella typhimurium.. J Bacteriol.

[41] Goodier RI, Ahmer BM (2001). SirA orthologs affect both motility and virulence.. J Bacteriol.

[42] Chakravortty D, Rohde M, Jager L, Deiwick J, Hensel M (2005). Formation of a novel surface structure encoded by Salmonella Pathogenicity Island 2.. EMBO J.

[43] Waterman SR, Holden DW (2003). Functions and effectors of the Salmonella pathogenicity island 2 type III secretion system.. Cell Microbiol.

[44] Espina M, Olive AJ, Kenjale R, Moore DS, Ausar SF (2006). IpaD Localizes to the Tip of the Type III Secretion System Needle of Shigella flexneri.. Infect Immun.

[45] Galan JE, Wolf-Watz H (2006). Protein delivery into eukaryotic cells by type III secretion machines.. Nature.

[46] Karavolos MH, Roe AJ, Wilson M, Henderson J, Lee JJ (2005). Type III secretion of the Salmonella effector protein SopE is mediated via an N-terminal amino acid signal and not an mRNA sequence.. J Bacteriol.

[47] Lucas RL, Lee CA (2001). Roles of hilC and hilD in regulation of hilA expression in Salmonella enterica serovar Typhimurium.. J Bacteriol.

[48] Ellermeier CD, Ellermeier JR, Slauch JM (2005). HilD, HilC and RtsA constitute a feed forward loop that controls expression of the SPI1 type three secretion system regulator hilA in Salmonella enterica serovar Typhimurium.. Mol Microbiol.

[49] Papezova K, Gregorova D, Jonuschies J, Rychlik I (2007). Ordered expression of virulence genes in Salmonella enterica serovar typhimurium.. Folia Microbiol.

[50] Winson MK, Swift S, Fish L, Throup JP, Jorgensen F (1998). Construction and analysis of luxCDABE-based plasmid sensors for investigating N-acyl homoserine lactone-mediated quorum sensing.. FEMS Microbiol Lett.

[51] Winson MK, Swift S, Hill PJ, Sims CM, Griesmayr G (1998). Engineering the luxCDABE genes from Photorhabdus luminescens to provide a bioluminescent reporter for constitutive and promoter probe plasmids and mini-Tn5 constructs.. FEMS Microbiol Lett.

[52] Karavolos MH, Spencer H, Bulmer DM, Thompson A, Winzer K (2008). Adrenaline modulates the global transcriptional profile of Salmonella revealing a role in the antimicrobial peptide and oxidative stress resistance responses.. BMC Genomics.

[53] Krell T, Lacal J, Busch A, Silva-Jimenez H, Guazzaroni ME (2010). Bacterial sensor kinases: diversity in the recognition of environmental signals.. Annu Rev Microbiol.

[54] Stock AM, Robinson VL, Goudreau PN (2000). Two-component signal transduction.. Annu Rev Biochem.

[55] Clarke DJ (2010). The Rcs phosphorelay: more than just a two-component pathway.. Future Microbiol.

[56] Pescaretti ML, Lopez FE, Morero RD, Delgado MA (2010). Transcriptional autoregulation of the RcsCDB phosphorelay system in Salmonella enterica serovar Typhimurium.. Microbiology.

[57] Arricau N, Hermant D, Waxin H, Ecobichon C, Duffey PS (1998). The RcsB-RcsC regulatory system of Salmonella typhi differentially modulates the expression of invasion proteins, flagellin and Vi antigen in response to osmolarity.. Mol Microbiol.

[58] Majdalani N, Gottesman S (2005). The Rcs phosphorelay: a complex signal transduction system.. Annu Rev Microbiol.

[59] Iwai N, Nagai K, Wachi M (2002). Novel S-benzylisothiourea compound that induces spherical cells in Escherichia coli probably by acting on a rod-shape-determining protein(s) other than penicillin-binding protein 2.. Biosci Biotechnol Biochem.

[60] Waidner B, Specht M, Dempwolff F, Haeberer K, Schaetzle S (2009). A novel system of cytoskeletal elements in the human pathogen Helicobacter pylori.. PLoS Pathog.

[61] Cowles KN, Gitai Z (2010). Surface association and the MreB cytoskeleton regulate pilus production, localization and function in Pseudomonas aeruginosa.. Mol Microbiol.

[62] Lober S, Jackel D, Kaiser N, Hensel M (2006). Regulation of Salmonella pathogenicity island 2 genes by independent environmental signals.. Int J Med Microbiol.

[63] Hensel M (2000). Salmonella pathogenicity island 2.. Mol Microbiol.

[64] Hensel M, Shea JE, Waterman SR, Mundy R, Nikolaus T (1998). Genes encoding putative effector proteins of the type III secretion system of Salmonella pathogenicity island 2 are required for bacterial virulence and proliferation in macrophages.. Mol Microbiol.

[65] Chiu S-W, Chen S-Y, Wong H (2008). Localization and Expression of MreB in Vibrio parahaemolyticus under Different Stresses.. Appl Environ Microbiol.

[66] Hormaeche CE (1979). Genetics of Natural-Resistance to Salmonellae in Mice.. Immunology.

[67] Shea JE, Beuzon CR, Gleeson C, Mundy R, Holden DW (1999). Influence of the Salmonella typhimurium pathogenicity island 2 type III secretion system on bacterial growth in the mouse.. Infect Immun.

[68] Vazquez-Torres A, Xu Y, Jones-Carson J, Holden DW, Lucia SM (2000). Salmonella pathogenicity island 2-dependent evasion of the phagocyte NADPH oxidase.. Science.

[69] Hoiseth SK, Stocker BA (1981). Aromatic-dependent Salmonella typhimurium are non-virulent and effective as live vaccines.. Nature.

[70] Murray RA, Lee CA (2000). Invasion genes are not required for Salmonella enterica serovar typhimurium to breach the intestinal epithelium: evidence that salmonella pathogenicity island 1 has alternative functions during infection.. Infect Immun.

[71] Beuzon CR, Salcedo SP, Holden DW (2002). Growth and killing of a Salmonella enterica serovar Typhimurium sifA mutant strain in the cytosol of different host cell lines.. Microbiology.

[72] Faulds-Pain, AK (2008). The Regulation of Flagellar Filament Assembly in Caulobacter Crecentus [dissertation].. Newcastle University.

[73] Bolivar F (1978). Construction and characterization of new cloning vehicles. III. Derivatives of plasmid pBR322 carrying unique Eco RI sites for selection of Eco RI generated recombinant DNA molecules.. Gene.

[74] Sutcliffe JG (1979). Complete nucleotide sequence of the Escherichia coli plasmid pBR322.. Cold Spring Harbor Symp Quant Biol 43 Pt.

[75] Guzman LM, Belin D, Carson MJ, Beckwith J (1995). Tight regulation, modulation, and high-level expression by vectors containing the arabinose PBAD promoter.. J Bacteriol.

[76] Hautefort I, Proenca MJ, Hinton JC (2003). Single-copy green fluorescent protein gene fusions allow accurate measurement of Salmonella gene expression in vitro and during infection of mammalian cells.. Appl Environ Microbiol.

[77] Gotfredsen M, Gerdes K (1998). The Escherichia coli relBE genes belong to a new toxin antitoxin gene family.. Mol Microbiol.

