# Supplementary figures and images for: The Bacterial Cytoskeleton Modulates Motility, Type 3 Secretion, and Colonization in Salmonella
Source: PLoS Pathog. 2012 Jan 26;8(1):e1002500. doi: 10.1371/journal.ppat.1002500 (PMC3266929; doi:10.1371/journal.ppat.1002500)

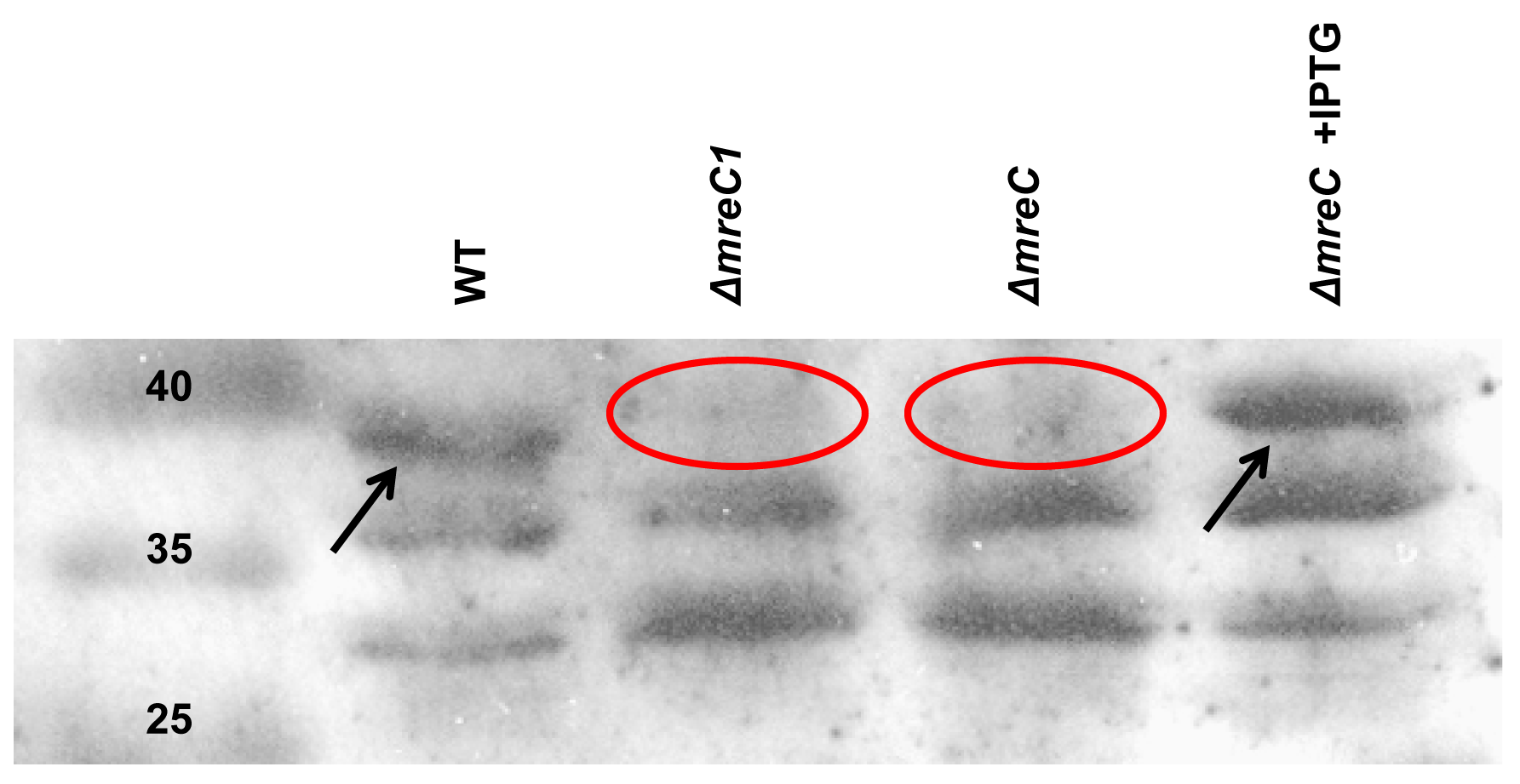

Supplement: Figure S1 — Expression of MreC in complemented Δ mreC cells. Western blot of total protein samples from SL1344 WT, ΔmreC1, ΔmreC, and ΔmreC plus 100 µM IPTG cells using αMreC antibody. MreC is indicated at approximately 38kDa and is distinguishable from background bands. (TIF) [file ppat.1002500.s001.tif]

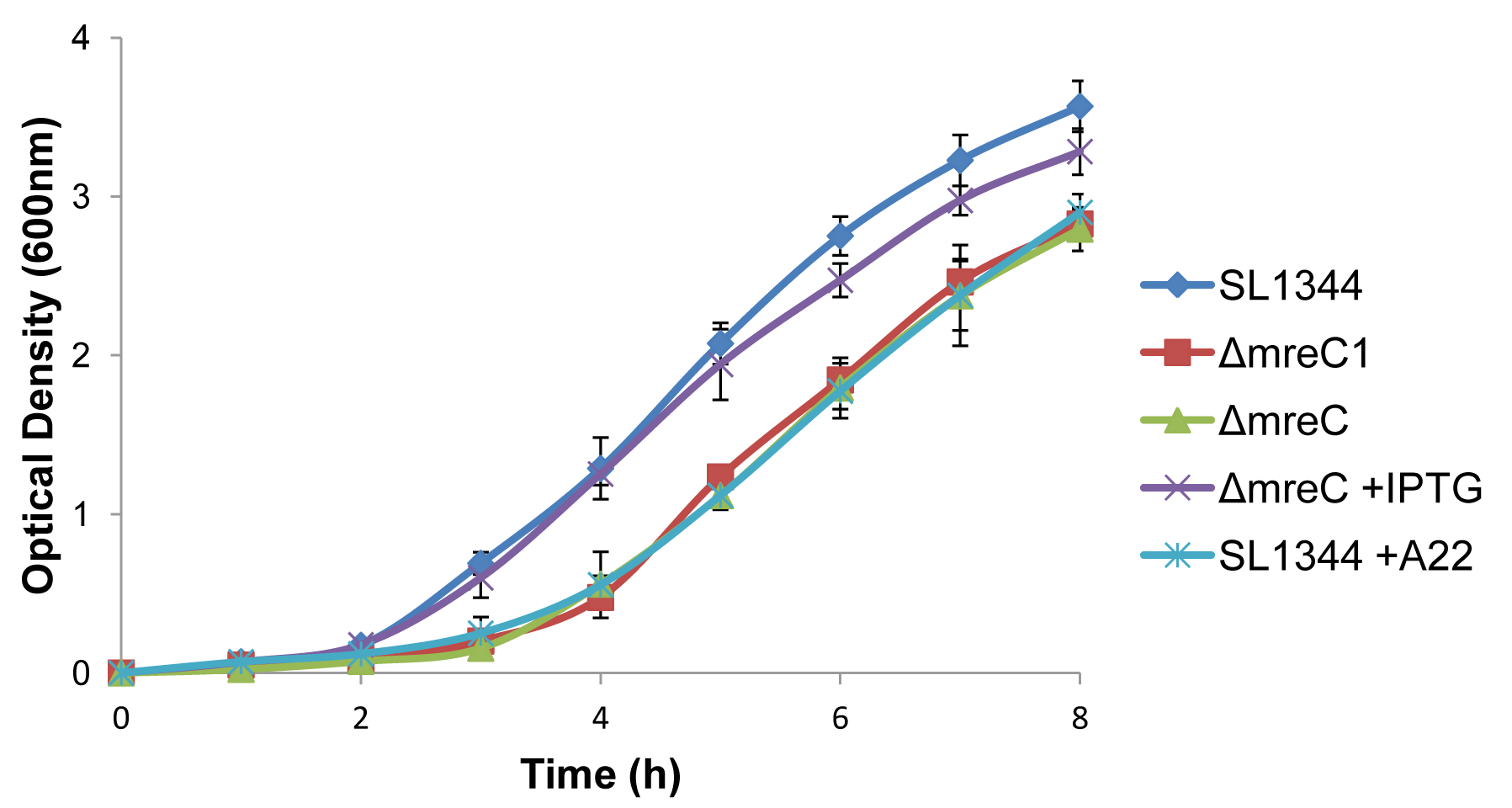

Supplement: Figure S2 — Growth curve of Salmonella mutant cells. Log phase growth of SL1344 WT, ΔmreC1, ΔmreC, ΔmreC plus 100 µM IPTG, and A22 treated SL1344 WT cells. Strains were grown in LB media at 37°C. (TIF) [file ppat.1002500.s002.tif]

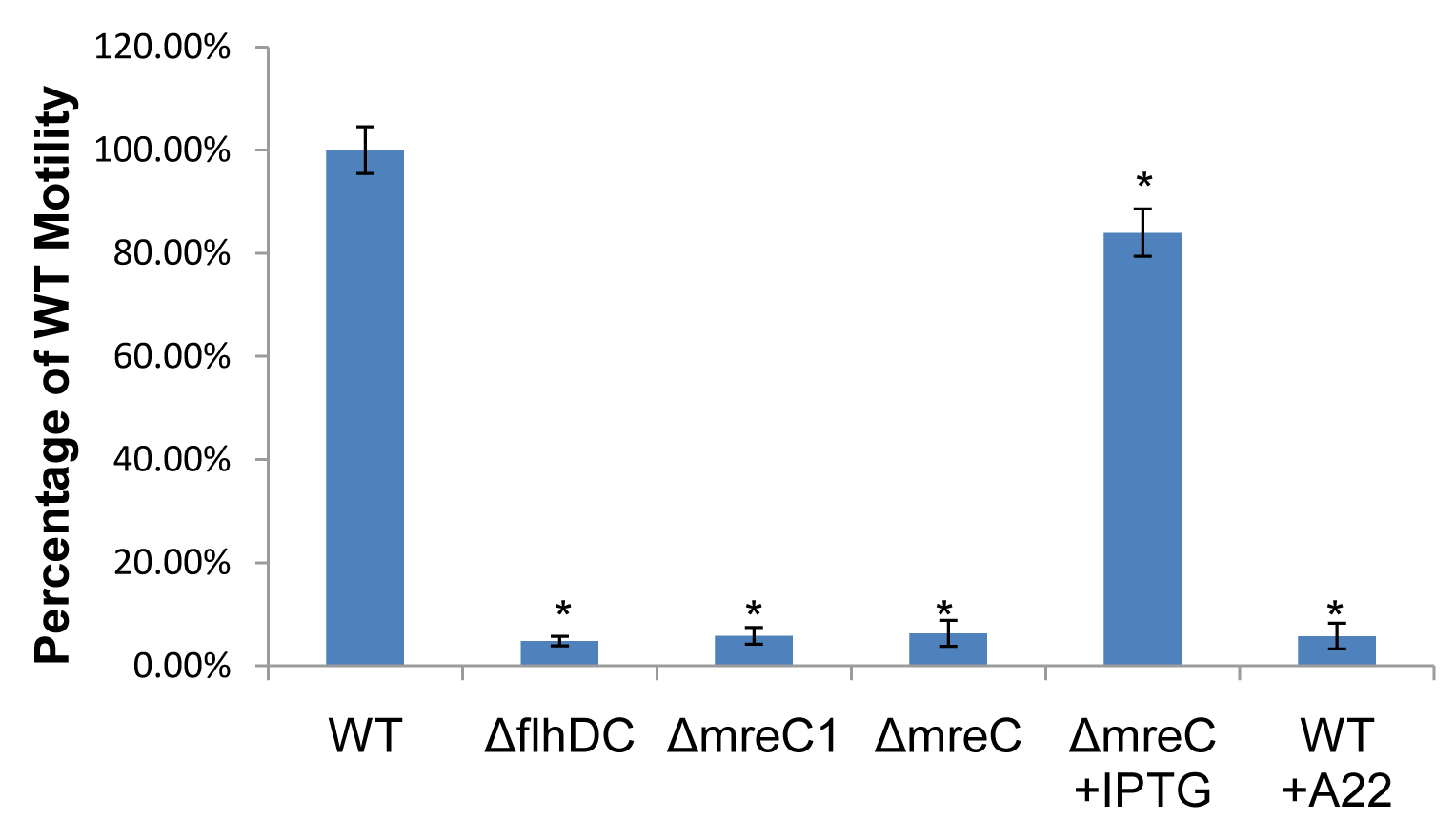

Supplement: Figure S3 — Motility of Salmonella Δ mre mutant cells. Motility of SL1344 WT, ΔflhDC, ΔmreC1, ΔmreC, ΔmreC plus 100 µM IPTG, and A22 treated SL1344 WT shown as a percentage of the wild type. Strains were grown on motility agar at 37°C. Experiments were repeated at least three times and error bars indicate SD. * Indicates statistical difference from WT (p<0.05). (TIF) [file ppat.1002500.s003.tif]

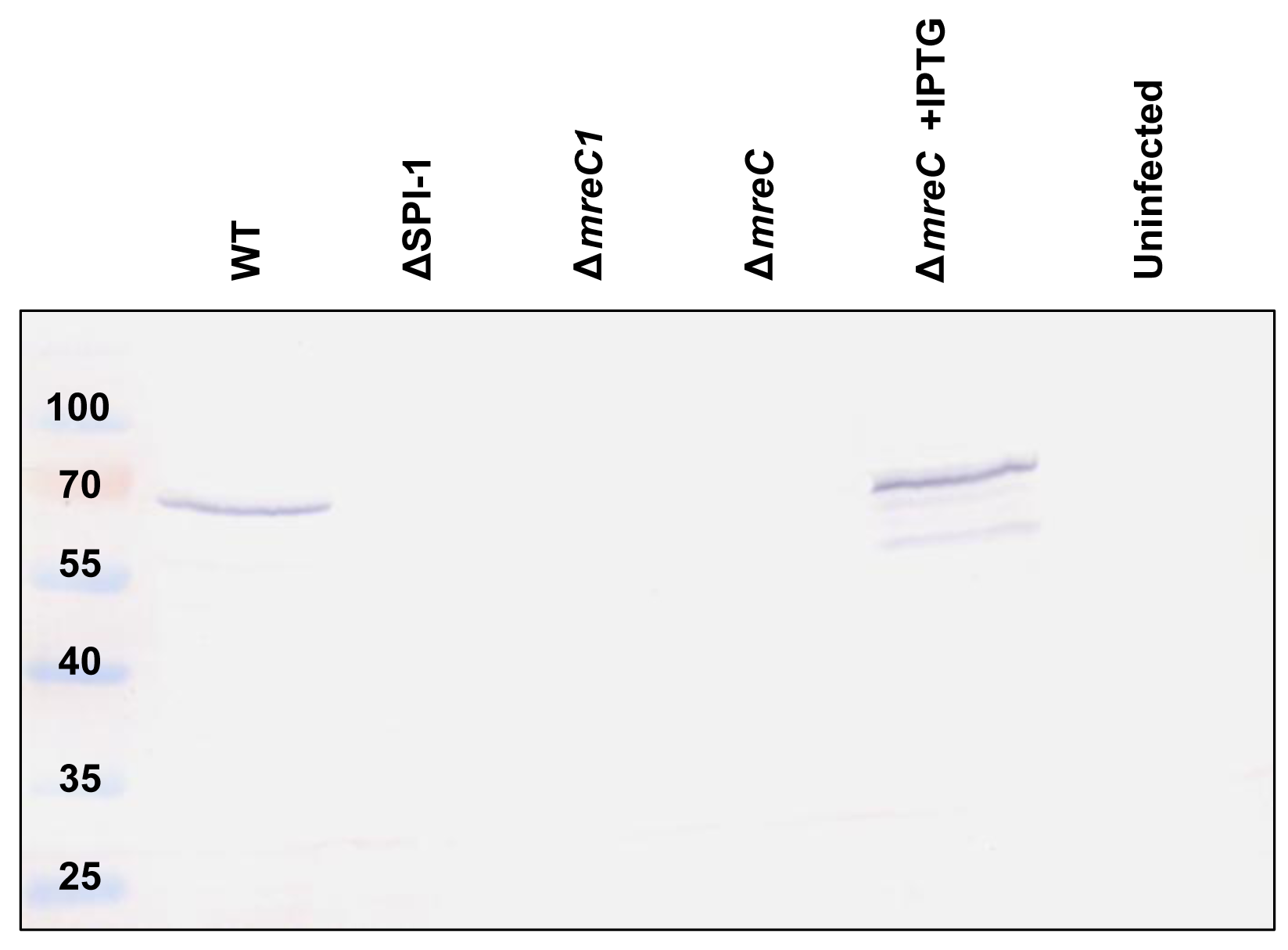

Supplement: Figure S4 — Translocation of SipB SPI-1 effector protein into Caco-2 cells. Western blot of host cytosol fractions with αSipB antibody following infection of cells with Salmonella SL1344 WT, ΔSPI-1, ΔmreC1, ΔmreC (+/− IPTG) mutants. SipB is indicated at approximately 65kDa. (TIF) [file ppat.1002500.s004.tif]

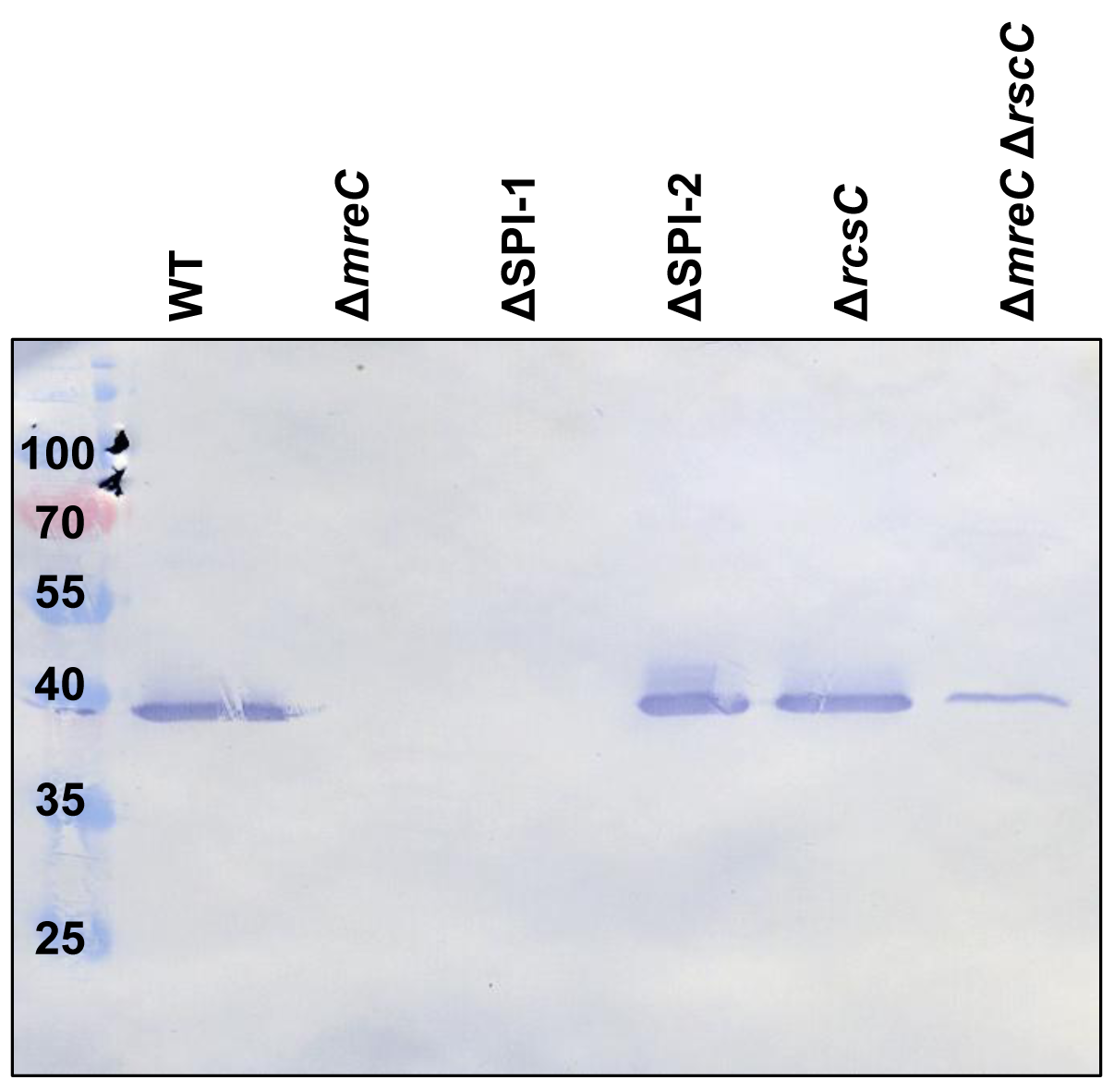

Supplement: Figure S5 — Secretion of SPI-1 effector protein SipC in Δ rcs C mutant cells. Western blot of secreted protein samples from SL1344 WT, ΔmreC, ΔSPI-1, ΔSPI-2, ΔrcsC, and ΔmreC ΔrcsC cells using αSipC antibody. SipC is indicated at approximately 43kDa. (TIF) [file ppat.1002500.s005.tif]

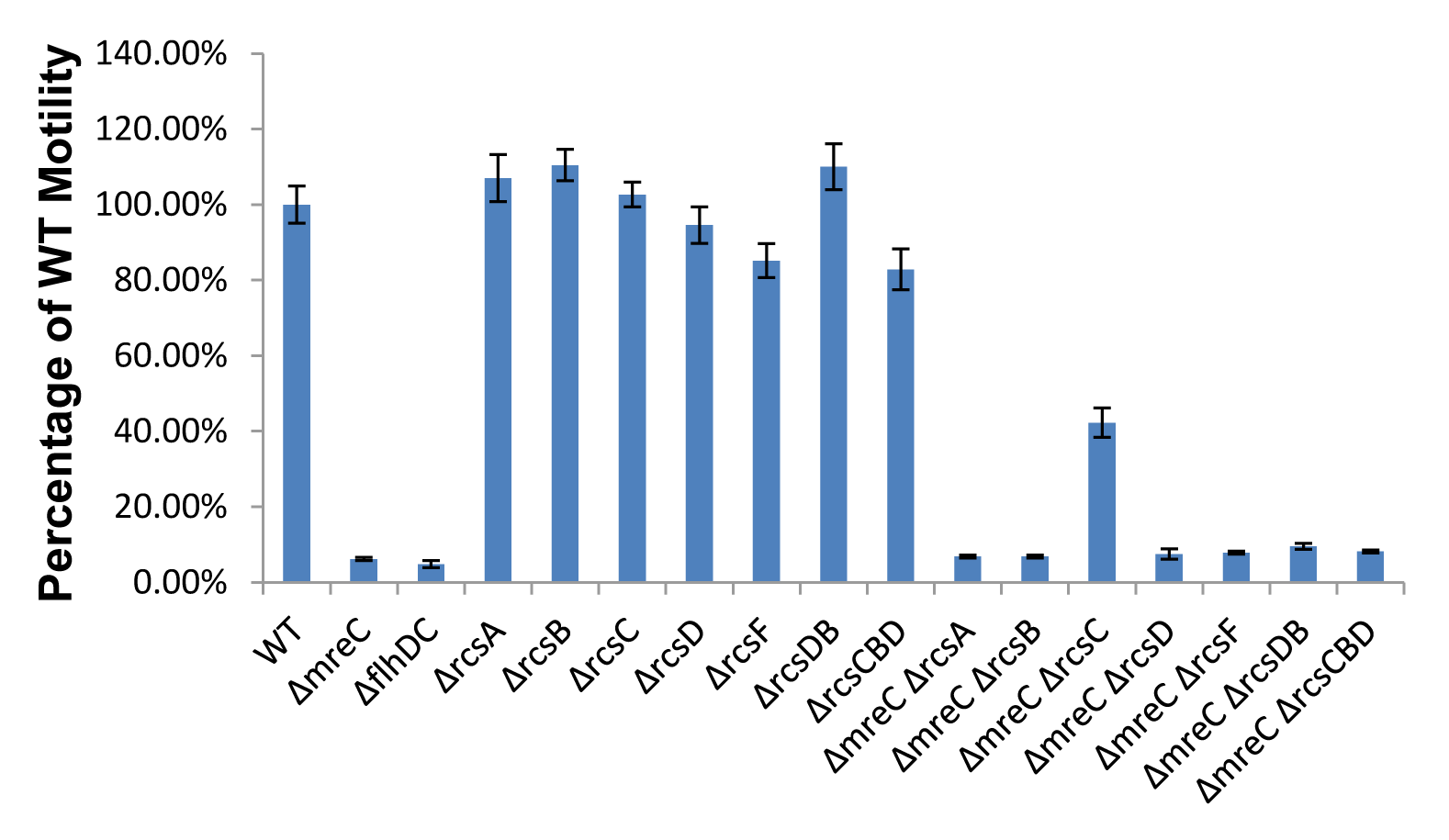

Supplement: Figure S6 — Motility of Salmonella Δ rcs mutant cells. Motility of SL1344 WT, ΔmreC, ΔflhDC, ΔrcsA, ΔrcsB, ΔrcsC, ΔrcsD, ΔrcsF, ΔrcsDB, ΔrcsCBD, ΔmreC ΔrcsA, ΔmreC ΔrcsB, ΔmreC ΔrcsC, ΔmreC ΔrcsD, ΔmreC ΔrcsF, ΔmreC ΔrcsDB, and ΔmreC ΔrcsCBD cells shown as a percentage of the wild type. Experiments were repeated at least three times and error bars indicate SD. Strains were grown on motility agar at 37°C. (TIF) [file ppat.1002500.s006.tif]

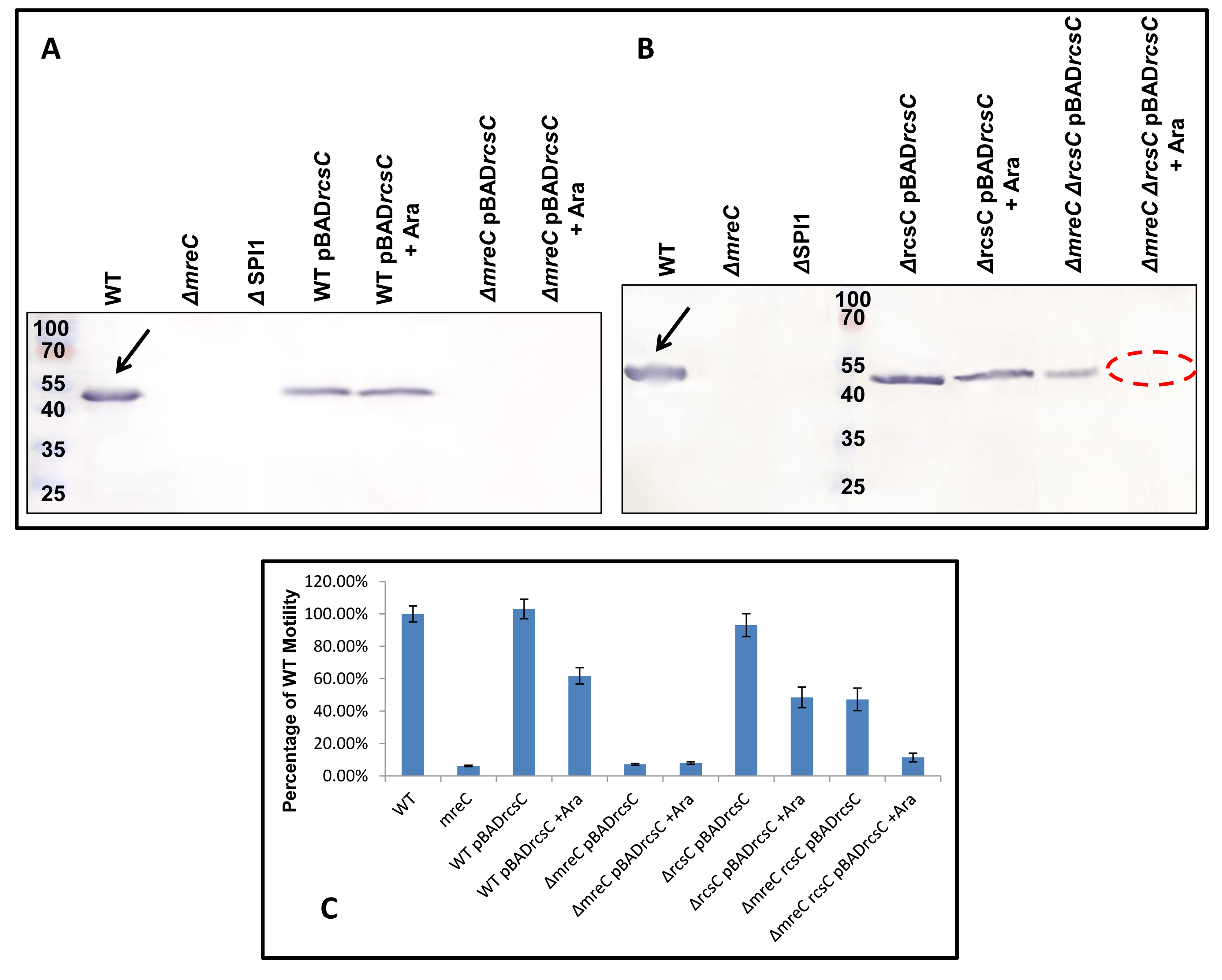

Supplement: Figure S7 — Effect of rcsC expression on SipC production and motility. Panels A and B show western blots from SL1344 WT, mreC, and SPI-1 control strains, and SL1344 WT pBADrcsC, mreC pBADrcsC, rcsC pBADrcsC, and mreC rcsC pBADrcsC strains (+/− arabinose) with αSipC antibody. SipC is indicated at approximately 43kDa. Panel C shows motility of SL1344 WT, mreC, SL1344 WT pBADrcsC, mreC pBADrcsC, rcsC pBADrcsC, and mreC rcsC pBADrcsC strains (+/− arabinose) shown as a percentage of the wild type. Experiments were repeated at least three times and error bars indicate standard deviation. (TIF) [file ppat.1002500.s007.tif]

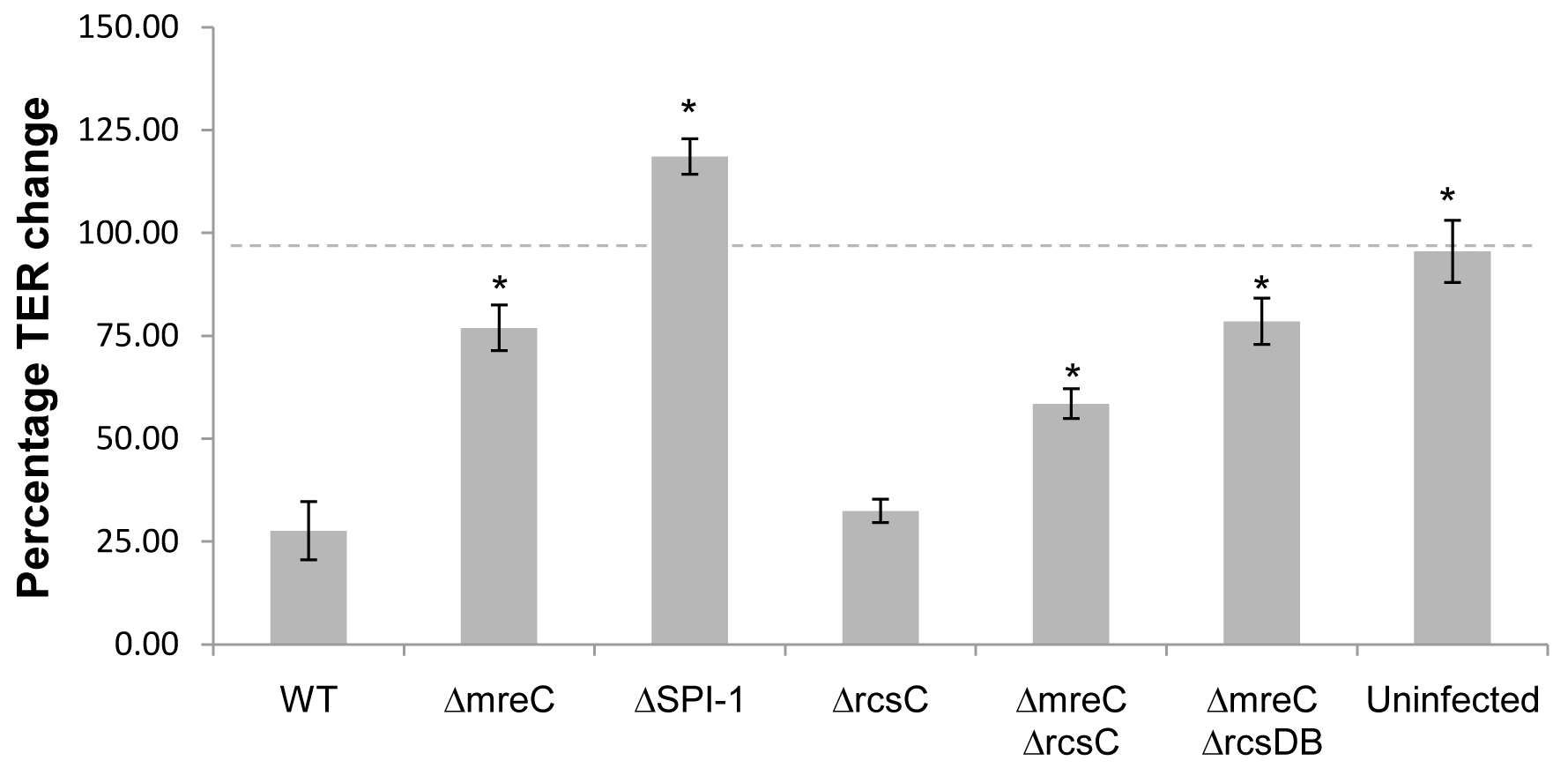

Supplement: Figure S8 — Percentage change in transepithelial resistance of differentiated Caco-2 cells after 4hr infection with Δ rcs mutant strains. TER of polarised Caco-2 monolayers exposed to Salmonella strains at an MOI of 20. TER change is expressed as a percentage alteration at 4hr compared to the initial value at time zero. Error bars indicate the standard deviations derived from at least three independent experiments. * Indicates statistical difference from WT (p<0.05). (TIF) [file ppat.1002500.s008.tif]

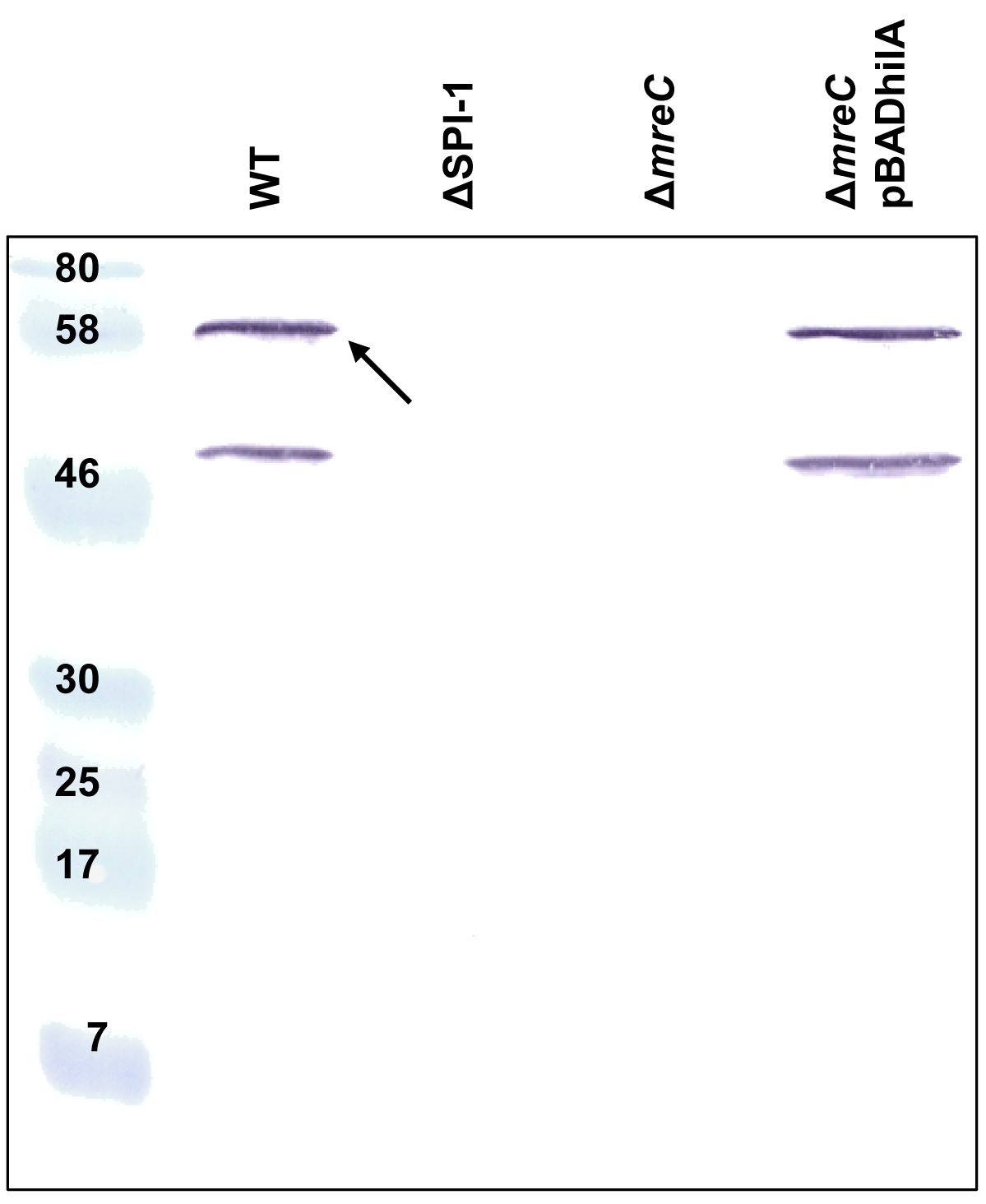

Supplement: Figure S9 — Complementation of Salmonella Pathogenicity Island SPI-1 in Δ mreC mutant. Expression of SPI-1 proteins in WT SL1344, ΔSPI-1, and ΔmreC mutants, and complemented ΔmreC pBADhilA strain during SPI-1 inducing conditions as revealed by western blotting with polyclonal αSipB antibody. SipB is indicated at approximately 63kDa, and a breakdown product is evident. (TIF) [file ppat.1002500.s009.tif]
